# Differential response to prolonged amoxicillin treatment: long-term resilience of the microbiome versus long-lasting perturbations in the gut resistome

**DOI:** 10.1080/19490976.2022.2157200

**Published:** 2022-12-28

**Authors:** Achal Dhariwal, Lars Christian Haugli Bråten, Kjersti Sturød, Gabriela Salvadori, Ahmed Bargheet, Heidi Åmdal, Roger Junges, Dag Berild, John-Anker Zwart, Kjersti Storheim, Fernanda Cristina Petersen

**Affiliations:** a Institute of Oral Biology, Faculty of Dentistry, University of Oslo, Oslo, Norway; b Department of Research and Innovation, Division of Clinical Neuroscience, Oslo University Hospital HF, Oslo, Norway; c Department of Infectious Diseases, Oslo University Hospital HF, Oslo, Norway; d Institute of Clinical Medicine, University of Oslo, Oslo, Norway; e Department of Physiotherapy, Faculty of Health Science, Oslo Metropolitan University, Oslo, Norway

**Keywords:** Antimicrobial resistance, microbiome, resistome, antimicrobial resistance gene, amoxicillin, antibiotics, chronic low back pain, Modic changes, functional metagenomics, shotgun

## Abstract

The collateral impact of antibiotics on the microbiome has attained increasing attention. However, the ecological consequences of long-term antibiotic exposure on the gut microbiome, including antibiotic resistance, are still limited. Here, we investigated long-term exposure effects to amoxicillin on the human gut microbiome and resistome. Fecal samples were collected from 20 patients receiving 3-months of amoxicillin or placebo treatment as part of a Norwegian multicenter clinical trial on chronic low back pain (AIM study). Samples were collected at baseline, last day of treatment, and 9 months after antibiotic cessation. The abundance and diversity of microbial and resistome composition were characterized using whole shotgun and functional metagenomic sequencing data. While the microbiome profiles of placebo subjects were stable over time, discernible changes in diversity and overall microbiome composition were observed after amoxicillin treatment. In particular, health-associated short-chain fatty acid producing species significantly decreased in proportion. However, these changes were short-lived as the microbiome showed overall recovery 9 months post-treatment. On the other hand, exposure to long-term amoxicillin was associated with an increase in total antimicrobial resistance gene load and diversity of antimicrobial resistance genes, with persistent changes even at 9 months post-treatment. Additionally, beta-lactam resistance was the most affected antibiotic class, suggesting a targeted response to amoxicillin, although changes at the gene level varied across individuals. Overall, our results suggest that the impact of prolonged amoxicillin exposure was more explicit and long-lasting in the fecal resistome than in microbiome composition. Such information is relevant for designing rational administration guidelines for antibiotic therapies.

## Introduction

Global health-care systems are under severe threat due to the increasing prevalence of antibiotic-resistant pathogens, causing treatment failure, higher mortality, and increased economic burden.^1,2^ Imprudent use of antibiotics is one of the significant drivers for the emergence and spread of antibiotic resistance.^3–6^ Increased consumption of antibiotics may not only affect us at the individual patient level but may also lead to greater resistance at regional, national and global levels.^7^

The advancements in next-generation sequencing technologies have led to an increased understanding of the gut microbiome and its role in modulating human health and physiology.^8,9^ Our gut microbiome is a complex ecosystem of microbial communities and an important well-known reservoir for a vast number of antibiotic resistance genes (ARGs). Moreover, it has been shown to contribute to the spread of resistance by promoting the horizontal gene transfer (HGT) or exchange of ARGs to opportunistic pathogenic bacteria.^10–13^ Since the human gut microbiome is a crucial player in the emergence and dissemination of antibiotic resistance, it is important to characterize the landscape of antibiotic resistance (resistome) in this microbial environment to contribute to personalized antimicrobial stewardship strategies.

Antibiotics can target not only the infective pathogen but also commensal and opportunistic bacteria inhabiting the human gut. Such collateral off-target effects can disrupt the composition or diversity of the gut ecosystem and are associated with significant health consequences.^14–17^ Antibiotic administration can also influence the gut microbiome by selecting antibiotic-resistant bacteria,^18^ increasing the abundance of particular ARGs^19,20^, and altering the resistome composition.^21,22^ Antibiotic exposure not only gives rise to resistance to the antibiotic used but also to other classes of antibiotics via cross-resistance or selection of co-localized genes that confer resistance to multiple antibiotics.^23,24^ In addition, the use of antibiotics also affects the dynamics of overall HGT, and thus enables the global spread of resistance by promoting the dissemination of ARGs located on mobile genetic elements (MGEs) within and between bacterial species.^25^ These disruptive effects on the microbiome and resistome can be transient or persistent, depending upon the antibiotics class, mode of action, dosage, duration, pharmacokinetic properties, and baseline taxonomic composition.^15,18,22,26–28^

Amoxicillin is one of the most commonly prescribed antibiotics worldwide, used to treat common bacterial infections in the ear, nose and throat.^29^ It is a broad-spectrum beta-lactam antibiotic that interferes with bacterial cell wall synthesis. So far, several culture and sequencing-based studies have shown that the adult human gut microbiome is generally resilient toward amoxicillin intervention, with little or no impact on its microbial composition.^30,31^ However, these studies are largely limited to taxonomic characterization and cannot elucidate the repertoire of ARGs, as they are mainly based on 16S rRNA gene amplicon sequencing. For identifying the landscape of antibiotic resistance (repertoire of ARGs) in microbiomes, whole metagenomic sequencing stands as the most powerful high-throughput method. This approach was recently used to assess the impact of 5-days amoxicillin therapy on the adult microbiome and its associated resistome. Although the results indicated a substantial enrichment of multiple classes of ARGs in the human gut due to amoxicillin treatment, most of the results were based on predictive analysis, and only one sample was analyzed using whole metagenomic sequencing.^32^ The impact of different class of antibiotics has also been examined, but primarily focusing on short-term (<7 days) treatments.^18,20–22,30,32,33^ For prolonged antibiotic therapy regimens, the effects on the human gut microbiome and resistome remain largely unknown.

Conditions for which prolonged amoxicillin treatment are generally used include recurrent otitis media, prophylaxis of urinary tract infection, and chronic respiratory conditions. Long-term amoxicillin treatment is also used to treat patients with chronic low back pain and vertebral endplate changes (Modic changes) visible on magnetic resonance imaging. The underlying rationale is a theory that Modic changes are often due to a low-grade bacterial infection.^34^ However, a recent multicenter, randomized, double-blind, placebo-controlled trial (the AIM study)^35^ concluded that the 3 months of amoxicillin use did not provide a clinically significant benefit in patients with chronic low back pain and Modic changes. Such prolonged antibiotic therapies are of special concern as the pressure in selecting and promoting the development of antibiotic resistance is expected to be higher than for short-term therapies.

In the present study, we have utilized next-generation deep shotgun sequencing and functional metagenomics to investigate the prolonged consequences of long-term antibiotic treatment on the gut microbiome and resistome of patients with chronic low back pain and Modic changes as compared to placebo. Here, we have examined the changes in the abundance, diversity and composition of microbial taxa and ARGs as well as explored the potential associations between them by analyzing the fecal samples from the subset of patients included in the AIM study. Overall, our results suggest that the impact of amoxicillin was severe yet short-lived in the microbiome composition compared to the changes in the resistome, which were more explicit and persistent, leading to a more diverse and abundant resistome even after 9 months post-antibiotic exposure.

## Materials and methods

### Ethics statement

The clinical trial was ethically approved by the regional Committees for Medical Research Ethics in Norway (2014/158/REK sør-øst) and the Norwegian Medicines Agency (SLV, reference number 14/01368-11, EudraCT Number: 2013–004505-14). It was registered at ClinicalTrials.gov (NCT02323412) on December 23, 2014. The risks and benefits of the study were explained, and written informed consent was obtained from all the participants.

### Study design and participants

To investigate the impact of prolonged broad-spectrum antibiotic (amoxicillin) exposure on the gut microbiome and resistome, we have included 20 patients who were randomly assigned to either amoxicillin (*n* = 8) or the placebo (*n* = 12) group (Table 1). The patients were recruited from a larger double-blind, placebo-controlled, randomized, multicenter trial (The AIM study), testing the efficacy of antibiotic treatment in 180 patients with chronic low back pain and Modic changes.^35^ The 20 selected patients were those enrolled at two of the study centers, Oslo University Hospital and Østfold Hospital, from April 2016 to September 2017. Fourteen patients did not provide samples for all time points and an additional two patients in the placebo group took antibiotics during the trial. These were excluded from the study. In this study, patients were relatively healthy adult human subjects aged between 27 and 62 y (mean ± SD: 45 ± 11), 15 (68%) were women, and their BMI ranged from 18 to 35 kg/m^2^ (mean ± SD: 24 ± 4). At baseline, 6 patients (27%) smoked while 8 (36%) were former smokers. All 20 patients in the amoxicillin group were compliant, i.e., took more than 80% of their study medication. Two patients in the amoxicillin group and four in the placebo group had gastroenteritis/diarrhea during the 3-month treatment period. The detailed inclusion and exclusion criteria for the main trial have already been described in the previously published protocol.^36^ In brief, participants had not taken any antibiotics 1 month before treatment intervention, nor did they travel abroad for a period exceeding 4 weeks. These patients were treated thrice a day with an oral dose of 750 mg amoxicillin or placebo for 3 months. All patients and research staff were blinded, and study medication was encapsulated to secure equal taste and smell. Fecal samples were collected at three time points: before the antibiotic treatment (baseline), post-treatment (3 months after the start of intervention), and 9 months (12 months from baseline) after the cessation of the treatment. In total, 60 fecal samples were collected in sterile tubes and stored at −80°C within 24 h until further processing and analysis. The basic metadata information for samples is also provided as Supplementary Table S8.

**Table 1.** Patient Demographics table.

		Amoxicillin (*n* = 8)	Placebo (*n* = 12)
Age, mean (SD)		41.0 (11)	46.6 (10)
Women (%)		7 (88%)	8 (67%)
BMI, mean (SD)		24.6 (4.7)	24.4 (4.2)
Smoking (%)			
	Current smokers	1 (13%)	5 (42%)
	Former smokers	4 (50%)	4 (33%)
	Never smoked	3 (38%)	3 (25%)
	missing	0	2 (17%)
Medications other than study medication (%)			
	Probiotics	6 (75%)	2 (17%)
	Lipid-lowering drugs	1 (13%)	1 (8%)
Adverse events (%)			
	Diarrhoea/gastroenteritis during 0–3 months’ follow-up	2 (25%)	4 (33%)
	Diarrhoea/gastroenteritis during 3–12 months’ follow-up	3 (38%)	1 (8%)

### DNA isolation and metagenomic sequencing

The microbial DNA from the fecal samples was extracted manually using the PSP® Spin Stool DNA Kit (Stratec molecular, Berlin, Germany) as per the manufacturer’s protocol. The quantity and quality of extracted DNA were accessed using a NanoDrop™ 2000c spectrophotometer (Thermo Fisher Scientific, Waltham, MA, USA), Qubit (Thermo Fisher Scientific) and agarose gel electrophoresis. Libraries for shotgun metagenomic sequencing were prepared using the Illumina Nextera Flex DNA library prep kit according to the manufacturer’s instructions. The DNA libraries were then sequenced with 150-nucleotide-long paired-end reads on an Illumina HiSeq 3000 platform (Illumina Inc., San Diego, CA, USA) at the Norwegian Sequencing Center (Oslo, Norway).

### Bioinformatics preprocessing

Low-quality and adapter sequences from paired-end reads were filtered using Trimmomatic^37^ (v.0.35) with the following parameters: ILLUMINACLIP: Nextera PE:2:30:10 LEADING:3 TRAILING:3 SLIDING WINDOW:4:15 MINLEN:36. After quality filtering, the human DNA contaminant sequences were discarded from all samples by filtering out the reads that mapped against the human reference genome (GRch38, downloaded from NCBI GenBank) using Bowtie2^38^ (v.2.3.4) with parameters q -N 1 -k 1 – fr – end-to-end – phred33 – very-sensitive – no-discordant. The quality of raw and clean reads was assessed using FastQC^39^ (v.0.11.8).

### Microbiome and resistome profiling

To investigate the microbial community composition, the clean, high-quality reads were subjected to taxonomic classification using MetaPhlAn 3.0^40^ containing ~17000 reference genomes of bacteria, archaea, eukaryotes and viruses. The resistome annotation of metagenomic reads was performed by mapping them against the nucleotide_fasta_protein_homolog_model from the Comprehensive Antibiotic Resistance Database (CARD)^41^ (v.3.0.9) antimicrobial resistance (AMR) database using Bowtie2 (v.2.3.4) with parameter – *very-sensitive-local*. Additionally, the ARGs that are not confidently expected to confer resistance based solely on a short-read marker were removed from further analysis as described by D’Souza et al.^42^ For annotation of ARGs, a coverage threshold of 80% (100% nucleotide homology with reference across 80% of target gene) was used. The mapped reads were filtered from unmapped reads, sorted and indexed using Samtools^43^ (v.1.9). The number of read counts mapped for each ARG was calculated using Bedtools^44^ (v.2.27.1). The counts were then normalized for differences in both gene lengths and bacterial abundances by calculating reads per kilobase reference per million bacterial reads (RPKM) values for every sample. The relative abundance of ARGs for each sample was estimated by dividing the RPKM by the sum of the RPKM for each sample. In addition to gene level, we also summed the RPKMs to higher functional levels, as annotated in their respective databases.

### Functional metagenomics

Three functional metagenomic libraries were constructed from pooled stool DNA of amoxicillin-treated patients, one for each time point, according to the published protocol,^45^ with minor modifications. In brief, each metagenomic library was cloned into pZE21 (kanamycin-resistant) and screened individually on Mueller–Hinton agar plates against 14 antibiotics (amoxicillin–clavulanate, aztreonam, carbenicillin, cefepime, cefoxitin, ceftazidime, colistin, meropenem, penicillin G, piperacillin, tigecycline, amoxicillin, ciprofloxacin and gentamicin), using the selective concentrations listed in Supplementary Table S2. All plates were incubated aerobically at 37°C for 24 h. In addition, a negative control plate of *E. coli* clones transformed with unmodified pZE21 (without metagenomic insert) was plated for each antibiotic selection to confirm that the concentration of antibiotic used completely inhibited the growth of clones with only pZE21. The functionally selected colonies were subsequently collected from the agar plate using an L-shaped cell-spreader and stored at – 80°C. To isolate the metagenomic inserts, the frozen stocks were then thawed, subjected to cell lysis, and pelleted by centrifugation at 15,000 × *g* for 2 min. The supernatants were then collected and used as a template for PCR amplification of metagenomic DNA fragments. These were further purified using the MinElute PCR Purification Kit (Qiagen) and prepared for sequencing using the NEXTFLEX® Rapid DNA-Seq Kit 2.0 (PerkinElmer).

The samples were sequenced on an Illumina NovaSeq platform (2 × 150 bp reads), and the quality of sequenced reads was accessed using FastQC. Similar to shotgun metagenomic data, quality filtering of sequenced reads was also performed using Trimmomatic with a slight change in the MINLEN:60 parameter. These quality-trimmed reads for each selection were assembled into contigs using metaSPAdes^46^ (v.3.13.0). Quality assessment of assembled contigs was done using MetaQUAST from QUAST^47^ (v.5.0.2) with (-m 1000) parameter. Notably, the selections were excluded from the analysis if the number of contigs assembled was more than tenfold of the number of colonies on the selection plate. Contigs with a length shorter than 500 bp were also filtered out. Finally, the open reading frames (ORFs) were predicted from the remaining contigs using the Prodigal^48^ (v.2.6.3) software. These ORFs were annotated hierarchically by searching them first against the BLAST-based (ResFinder,^49^ CARD,^41^ AMRFinder-Prot^50^) ARG databases, and then the residual ORFs were annotated using HMM-based (AMRFinder-hmm,^50^ Resfams^51^) ARG databases using publicly available *resAnnotator.py* pipeline (https://github.com/dantaslab/resAnnotator). The objective for such sequential annotation of ARGs was to first identify perfect hits to known resistance determinants through BLAST-based databases (with a high percent identity (>95%) and coverage (>95%) threshold) and afterward detect the variants of known resistance determinants using HMM-based databases. Further, FASTA files of all the annotated ARG sequences from all antibiotic selections were concatenated and perfectly identical ARGs were merged into one sequence using CD-HIT^52^ (v.4.8.1) with options: -c 1.0 -aS 1.0 -g 1 -d 0. The percent identity of all ARGs was determined using BlastP query against both a custom database that combined all ARG proteins from CARD and AMRFinder, and the database provided by NCBI for non-redundant proteins (nr database, retrieved January 25, 2022). The best local hit identified was then used for a global alignment using the needle program with the following non-default parameters: -gapopen-10 -gapextend = 0.5. The detailed protocol on functional validation of novel resistance gene along with its amino acid and nucleotide sequence is presented in Additional file S2.

The putative MGEs were detected from functional metagenomic assemblies through string searches to one of the following keywords in their Pfam and TIGRFAMs annotations: ‘transposon’, ‘plasmid’, ‘transposase’, ‘integron’, ‘conjugative’, ‘integrase’, ‘recombinase’, ‘conjugal’, ‘mobilization’ or ‘recombination’, as previously described.^53^

### Statistical analysis and data visualization

Statistical analysis was accomplished in R (v.3.6.4). The graphical illustrations were mainly made using ggplot2 (v.3.3.5) R package with post-editing in Adobe Illustrator (v.16.0.0). Diversity analysis (α- and β-diversity) were performed using the vegan (v.2.5.7) and phyloseq (v.1.34.0) packages. The changes in α-diversity over time points in groups were statistically evaluated using lnear mixed-effects (LME) model and Tukey’s HSD post hoc test using R nlme package. β-Diversity was conducted on centered log-ratio (CLR) transformed species and ARG relative abundance data, and principal component analysis (PCA) ordination technique was used to further understand which metadata variables were associated with the dispersion of samples microbiome and resistome compositions, respectively. Permutational multivariate analysis of variance (PERMANOVA) test by adonis function (vegan R package) was used to determine the statistical significance of β-diversity. The homogeneity of multivariate dispersion between treatment groups and time points was evaluated prior by permutational analysis of multivariate dispersion (PERMDISP) test with function betadisper (vegan). The dispersion of the data was homogenous at all time points within the treatment groups. Patient_ID was used as a random effect or strata in the models to account for interindividual variation present in the dataset. The compositional shifts were quantified and compared across individuals or pairs using Bray–Curtis and Jaccard distance metrices. The Jaccard distance accounts for the presence/absence of features (species or ARGs) and is thus more sensitive to changes driven by rare features. While Bray–Curtis distance accounts for differences in the relative abundance of features. For comparisons of group differences, Wilcoxon test using wilcox.test function was applied with paired = T/F, as appropriate. Differentially abundant features between time points within treatment groups for all the datasets were identified using LEfSe^54^ algorithm. Enterotypes were identified using the Dirichlet multinomial mixture (DMM) models approach using the DirichletMultinomial (v.1.38.0) package. Procrustes analysis was performed to determine the association or effect of underlying bacteriome on the resistome. The symmetric Procrustes correlation coefficients and significance were retrieved through the ‘protest’ function from the vegan package with 999 permutations. Heatmaps and synteny maps were generated using the Pheatmap (v.1.0.12) and Gggenes (v.0.4.1) R package, respectively. The *P*-values below or equal to 0.05 were considered significant and corrected for false discovery rate (FDR) where appropriate using Benjamini–Hochberg (BH) method.

## Results

### Detrimental short-term alterations in the diversity and composition of the human gut microbiome upon prolonged amoxicillin therapy

To examine the post-treatment and long-term taxonomic response to prolonged amoxicillin exposure, total DNA was extracted from fecal samples and sequenced on Illumina platform, resulting in ~200 Gb of raw sequencing data. From 60 fecal samples, we obtained a total of ~1.5 billion raw sequences with a number of reads ranging from 11.3 to 41.6 million (M) per sample with an average of 25.1 M reads. Overall, 49.5% (0.72 billion in total) of high-quality clean sequences with a number of reads ranging from 5.07 to 23.2 M per sample with an average of 12 M reads were classified as bacterial (Supplementary Table S1). In most of the samples, Bacteroidetes (mean 65.31%; SD 15.75%) and Firmicutes (mean 29.53%; SD 13.94%) were the most abundant phyla, followed by Actinobacteria (mean 2.94%; SD 3.57%) and Proteobacteria (mean 1.72%; SD 4.22%). These four phyla had a combined relative abundance of 99.5% across samples. At genus level, the microbiome composition was primarily dominated by *Bacteroides* (mean 39.06%; SD 17.68%), with *Prevotella* (mean 11.04%; SD 22.40%) and *Alistipes* (mean 7.87%; SD 6.37%) being the second and third most abundant genera. *Parabacteroides, Eubacterium, Blautia, Roseburia* and *Faecalibacterium* were the other most prevalent genera identified in all the patients at all time points. In total, 118 different genera (range: 41–71 per patient) were identified, with 16 common genera present across all patients.

**Figure 1.**
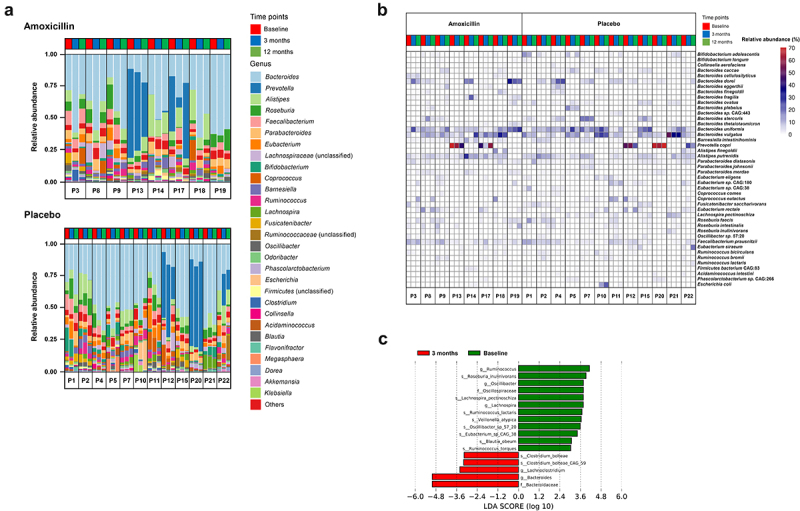
Composition of bacterial communities in all patients before exposure (baseline), 3 months later and 9 months after cessation of treatment (i.e., 12 months). (a) Bar plot representing the relative abundance of top 30 most abundant bacterial genera in amoxicillin (top) and placebo-exposed (bottom) participants. Genera with lower abundance were merged into “Others” category. Source Data: Supplementary Table S6. (b) Heat map representing the relative abundance of bacterial species that constituted >1% of the community in at least two samples. P precedes the identification number of the patient. (c) Histogram of LDA scores of bacterial taxa that were significantly different in abundance between baseline and 3 months in amoxicillin group identified using linear discriminant analysis (LDA) combined with effect size (LEfSe) algorithm, are shown, with a cutoff value of LDA score (log10) above 2.0. Bacterial taxa enriched in baseline are indicated with a positive LDA score (green) and taxa enriched immediately after the cessation of treatment (3 months) are represented by a negative score (red).

The overall individual microbial profiles did not differ substantially following antibiotic exposure, and most changes observed varied from individual to individual (Figure 1a & Supplementary Table S6). However, a common response to amoxicillin was observed in the treatment group. Indeed, the genera *Ruminococcus, Oscillibacter* and *Lachnospira* were decreased considerably in their relative abundances after amoxicillin intake, while the proportion of *Bacteroides* and *Lachnoclostridium* increased significantly in majority of the individuals. At higher taxonomic levels, we also observed changes in the proportion of two bacterial families, i.e., Bacteroidaceae and Oscillospiraceae, upon amoxicillin treatment (Supplementary Figure 1). In contrast, no substantial changes at genus and higher taxonomic levels were identified in the overall gut microbiome composition between any time points in the placebo group, suggesting the relative stability of the gut microbiome without antibiotic perturbations. Despite differences, enterotyping analysis using Dirichlet multinomial mixture (DMM) model dichotomized all the fecal samples into *Bacteroides* and *Prevotella* dominant genera based on the similarities in the composition of gut microbiome (Supplementary Figure 2).

The dominant species detected in the gut microbiome were typically associated with 1 of the 15 species belonging to the *Bacteroides, Faecalibacterium, Prevotella, Ruminococcus, Eubacterium, Roseburia, Alistipes, Coprococcus* and *Parabacteroides* genera (Figure 1b). *Faecalibacterium prausnitzii, Bacteroides vulgatus, Bacteroides uniformis, Alistipes putredinis and Parabacteroides distasonis* were among the most common and abundant species found across most of the patients. At the same time, some of the other bacterial species such as *Prevotella copri, Bacteroides dorei and Bacteroides stercoris* were predominantly present in some fecal samples. These findings were coherent with previously published studies, indicating the existence of a global core gut microbiome.^55^ Nevertheless, the changes in the microbiome composition at the species level were mostly specific for each individual, and no major changes in the dominant species were observed after exposure to antibiotics.

**Figure 2.**
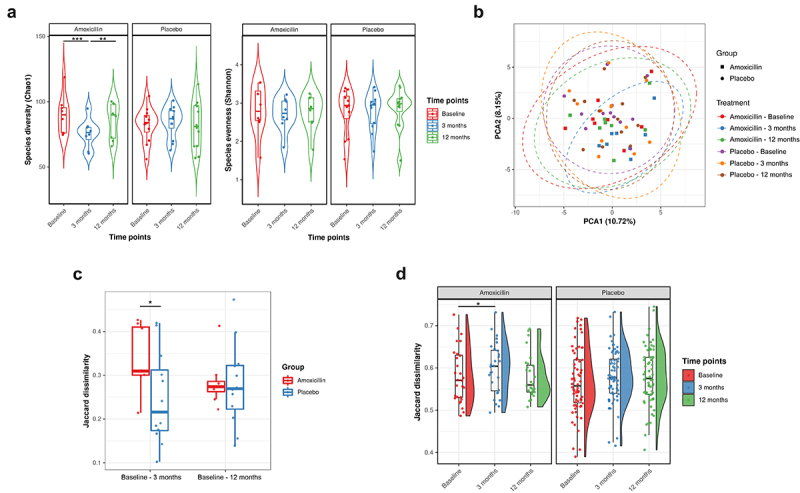
Impact of amoxicillin on fecal microbiota diversity and composition. (a) Violin plots reporting the species α-diversity measured and compared over time points in amoxicillin and placebo groups using Chao1 (richness; left) and Shannon (evenness; right) index, respectively. Each point corresponds to a given sample, and each box span from the first to third quartiles with a horizontal line inside the boxes representing the median. Adjusted *P* values were computed using the LME mixed effect model and the Tukey’s HSD post hoc test. Adjusted *P* values (*P*): ***P < .001, **P < .01; *P < .05. (b) Principal component analysis (PCA) based on the centered-log ratio (clr) transformed species abundance matrix. Each point represents the bacterial microbiome of an individual sample. Different symbols indicate different treatment groups; colors indicate different time points in different treatment groups. Ellipses represent 95% confidence intervals (CI) around the group clustered centroid. (c) Dissimilarity in microbiome composition between the baseline and the other time points, i.e., 3 and 12 months. Each point corresponds to Jaccard dissimilarity calculated between baseline and each of the other time point samples of the respective individual. Black horizontal line on the top connects statistically significantly different groups within each visit pair (*P < .05; Wilcoxon rank-sum test). (d) β-Diversity boxplots showing the distribution of the Jaccard dissimilarity in microbiome profile between individuals at the same time point within each treatment group. Each point is a comparison between two samples within the same time point group. The distributions are displayed to the right of the points, and boxplots showing the median and interquartile ranges are superimposed on top of the points (Statistical significance: paired Wilcoxon test (*P* values (*P*): *P < .05, **P < .01 and ***P < .001).

Next, we characterized the alteration in α-diversity using richness (Chao1) and evenness (Shannon) measures. Amoxicillin administration led to a significant reduction in species richness immediately after treatment cessation (3 months) as compared to placebo (LME: adj *P* = 1.94e-05), whereas evenness was not significantly reduced (adj *P* = .20). The species richness returned to baseline levels after 9 months post-treatment (Figure 2a). Contrarily, no significant alteration in Chao1 and Shannon diversity index was observed in the placebo group at all time points, revealing the overall stability of α-diversity over time.

Then, to assess the overall changes in the microbial community composition and structure, β-diversity analysis using principal component analysis (PCA) ordinate with Euclidean distance was conducted. PCA plot reveals a significant shift in the microbiome composition immediately after cessation of amoxicillin intervention (3 months) (PERMANOVA: *R*^2^ = 0.070; *P* = .007) (Figure 2b). However, the microbial shifts were not discernible after 9 months post-treatment (PERMANOVA: *R*^2^ = 0.02; *P* = .25). In addition, PERMANOVA analysis showed that the inter-individual differences in microbiome profile is greater than the effect of antibiotics as the samples from same individual tend to primarily cluster together (Supplementary Figure 3A, PERMANOVA: *R*^2^ = 0.79; *P* = .0001; *nperm* = 999). No significant alterations in the overall bacterial community composition were observed at any time point in the placebo group.

Microbial compositional shifts between the baseline and each of the other two time-point samples within each individual were quantified using Jaccard distance to determine the extent of alteration upon amoxicillin intervention. We observed that the average compositional differences between baseline and 3-month samples were significantly larger in the amoxicillin than in the placebo group (0.33 vs. 0.24, respectively, *P* = .042, Wilcoxon rank-sum test) (Figure 2c), though the composition in the amoxicillin group did not differ significantly from the placebo group at 12 months (0.28 vs. 0.27, respectively, *P* = .73, Wilcoxon rank-sum test).

We also investigated whether the gut microbiome presents a more common than an individualized response to amoxicillin treatment by targeting similar sets of species and converging the microbiome profile across individuals. In such case, we would expect the microbiome profiles to become more similar after amoxicillin treatment across individuals as a result of the selective stress imposed by antibiotics. However, the results from our analysis revealed that the average dissimilarity of the microbiome composition measured by the Jaccard distance between individuals increased significantly immediately after the amoxicillin treatment (0.57 at baseline vs. 0.61 at 3 months, *P* = .047, Wilcoxon signed rank exact test), but 9 months after the cessation of the amoxicillin, microbiome profiles converged to baseline levels (Figure 2d). No significant increase in microbiome divergence was observed in the placebo group over time. In general, these findings indicate that the overall composition of the microbiome diversified due to prolonged amoxicillin exposure, and that such effect persisted only for a short term.

Lastly, we explored the effect of treatment on individual microbial taxa. In concordance with the results regarding the alteration in richness and composition described above, significant differences were observed among species with low abundance. At the species level, well-known short-chain fatty acids (including butyrate) producers, such as *Roseburia inulinivorans, Ruminococcus torques, Lachnospira pectinoschiza, Ruminococcus lactaris, Veillonella atypica* and *Blautia obeum* were significantly reduced immediately after exposure to amoxicillin as compared to baseline (LEfSe). More recently discovered, “healthy gut” marker bacteria, i.e., *Oscillibacter* sp. *57*_*20*, was also found to be differentially decreased in abundance after cessation of treatment. Most of these species belong to phylum Firmicutes and class Clostridia (except *Veillonella atypica*) and are part of the normal gut microbiome. They play a crucial role in maintaining homeostasis, inflammation and oxidative stress in the human gut. However, all these species except *Lachnospira pectinoschiza* almost returned to their baseline abundance levels at 9 months post-treatment. In contrast, two species associated with overall poor gut health, i.e., *Clostridium bolteae* and *Clostridium bolteae CAG 59,^56^* were increased in most individuals exposed to amoxicillin and returned to baseline at the 12 months sampling time point (Figure 1c, Supplementary Figure 1 & Table S3). However, for *C. bolteae CAG 59, but not C. bolteae*, we also observed an increase in its proportion for some of the patients (4 out of 12) in the placebo group. In general, the microbiome composition in the placebo group remained stable, as no other microbial taxa were significantly increased or decreased over time.

### Long-term changes in the abundance and diversity of the gut resistome upon prolonged amoxicillin therapy

**Figure 3.**
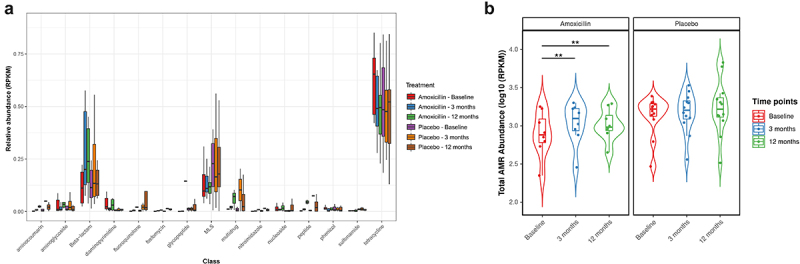
Beta-lactam and total AMR gene abundance increases after amoxicillin exposure. (a) Box plots showing the relative abundance measured as reads per kilobase million (RPKM) of antibiotic resistance gene (ARG) classes across all samples, stratified by treatment group and time points. The center horizontal line of box is median, box limits are upper and lower quartiles, whiskers are 1.5× interquartile ranges. (b) The total abundance, measured as the total reads per kilobase million (RPKM): Violin plots showing the total AMR genes abundance level per sample (represented as point), stratified by treatment and time points. The horizontal box lines represent the first quartile, the median and the third quartile. Whiskers denote the range of points within the first quartile − 1.5× the interquartile range and the third quartile + 1.5× the interquartile range. The black horizontal line on the top of plot connects statistically significantly different time points within each treatment group (Adjusted *P* values (P): ***P < .001, **P < .01; *P < .05; one-way ANOVA with repeated measures followed by post hoc pairwise *t*-test is used to check the statistical significance).

**Figure 4.**
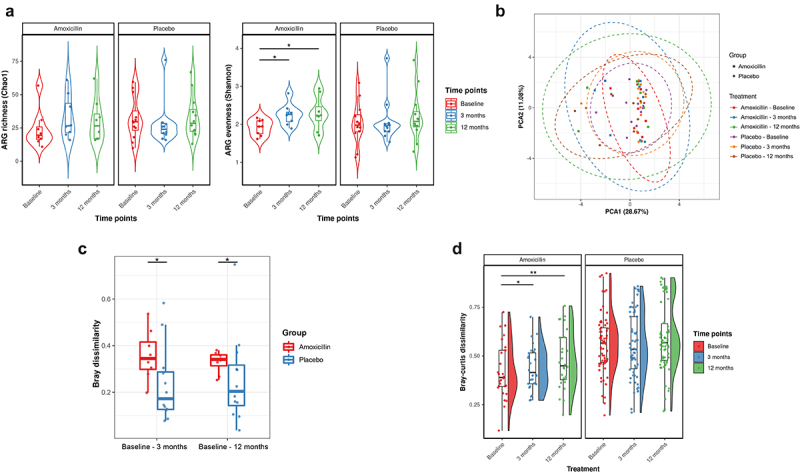
Impact of amoxicillin on fecal ARG diversity and composition. (a) Violin plots showing the ARG α-diversity measured and compared over time points in amoxicillin and placebo groups using Chao1 (richness; left) and Shannon (evenness; right) index, respectively. Each point corresponds to a given sample, and each box span from the first to third quartiles with a horizontal line inside the boxes representing the median. Adjusted *P* values were computed using the LME mixed effect model and Tukey’s HSD post hoc test. Adjusted *P* values (*P*): ***P < .001, **P < .01; *P < .05. (b) β-diversity was analyzed using principal component analysis (PCA) based on the clr-transformed ARG count abundance matrix. Each point represents a single sample, shape indicate treatment group, colored according to different time points and groups. Ellipses indicate 95% confidence intervals (CI). (c) ARG β-diversity measured by Bray–Curtis dissimilarity is compared between the amoxicillin (red) and placebo (blue) samples at baseline and the other time points, i.e., 3 and 12 months. Each point represents the dissimilarity in one individual’s resistome at baseline compared each of the other time point samples. Center line is median, box limits are upper and lower quartiles, whiskers are 1.5× interquartile ranges and points beyond whiskers are outliers (*P* < .05; Wilcox rank sum test). (d) Distribution of the Bray–Curtis distance of resistome (ARGs) between patients at the same time point within treatment groups. The statistical difference between the timepoints in both groups was tested by the paired Wilcoxon test, and the significance is marked with * *P* < .05, **P < .01 and ***P < .001.

In total, we detected 147 unique ARGs conferring resistance to 15 classes of antibiotics via 5 resistance mechanisms in the fecal microbiome of all the studied participants using shotgun metagenomic data (Supplementary Table S7). Tetracycline (mean 64.59%; SD 18.47%), beta-lactam (mean 14.10%; SD 13.45%), macrolide-lincosamide-streptogramin (MLS) (mean 12.48%; SD 13.40%), aminoglycoside (mean 1.78%; SD 6.35%) and multidrug (mean 2.36%; SD 6.35%) were the most abundant class of ARGs across samples, with the *tetQ, tetW, tetO, cfxA, mef(En2) and ermF* genes comprising the majority of alignments within these classes (Figure 3a & Supplementary Figure 4). The total AMR abundance measured as the total reads per kilobase million bacterial reads (RPKM) of all ARGs in the gut was lower in the amoxicillin than in the placebo group at baseline. Notably, the total AMR abundance significantly increased over time after amoxicillin treatment, suggesting that antibiotic treatment may enrich the microbiome for AMR determinants (Figure 3b). This increase in abundance corresponded with the increase in α-diversity evenness (Shannon) of ARGs in the resistome at 3-month timepoint (LME: adj *P* = .02), which remained significantly higher after 9 months post-treatment (LME: adj *P* = .01). A similar increase in ARG richness (Chao1) was observed after amoxicillin exposure, though the trend was not statistically significant. In contrast, no significant differences in the total ARG abundance and α-diversity measures were observed over time in the placebo group (Figure 4a).

Similar to the microbiome, the β-diversity PCA plot also showed that variation between different individuals (inter-individual variation) is the most dominant factor affecting the resistome composition (PERMANOVA: *R*^2^ = 0.82, *P* = .0001; *nperm* = 999; Supplementary Figure 3B). Moreover, the inter-individual variability of resistome was even higher than in microbiome composition, with most ARGs observed in a limited number of participants. At the same time, no visible pattern of resistome profile changes over time was observed in both placebo and treated groups (Figure 4b). However, PERMANOVA analysis with Bray–Curtis distance demonstrated a significant shift in the overall resistome composition after amoxicillin exposure at 3 months (PERMANOVA: Bray: *R*^2^ = 0.072; *P* = .046; strata = Patient) and 12 months (PERMANOVA: Bray: *R*^2^ = 0.074; *P* = .01; strata = Patient) when taking significant interindividual variation into account. Further, the compositional differences (Bray) observed upon amoxicillin exposure were higher and long-lasting, which remained statistically significant compared to the placebo group up to 12 months (Figure 4c; Wilcoxon rank-sum test), unveiling persistent diversification of resistome composition after antibiotic treatment. We also found that amoxicillin exposure was associated with prolonged resistome diversification through an individualized selection of ARGs as the dissimilarity of the resistome composition among the individuals significantly increased over time (Figure 4d; Wilcoxon signed rank exact test). When considering ARGs presence/absence rather than the abundance, similar resistome divergence was observed in the amoxicillin treatment group. In contrast, we observed that the resistome composition significantly converged at 3 months based on Jaccard distance in the placebo group (Supplementary Figure 5; Wilcoxon signed rank exact test). No significant divergence or convergence based on Bray–Curtis distance was detected in the resistome composition over time among placebo-treated individuals. Thus, these long-term changes observed in the resistome profiles may have resulted from a drug-specific selection rather than from short-term shifts discerned in the microbiome profiles.

**Figure 5.**
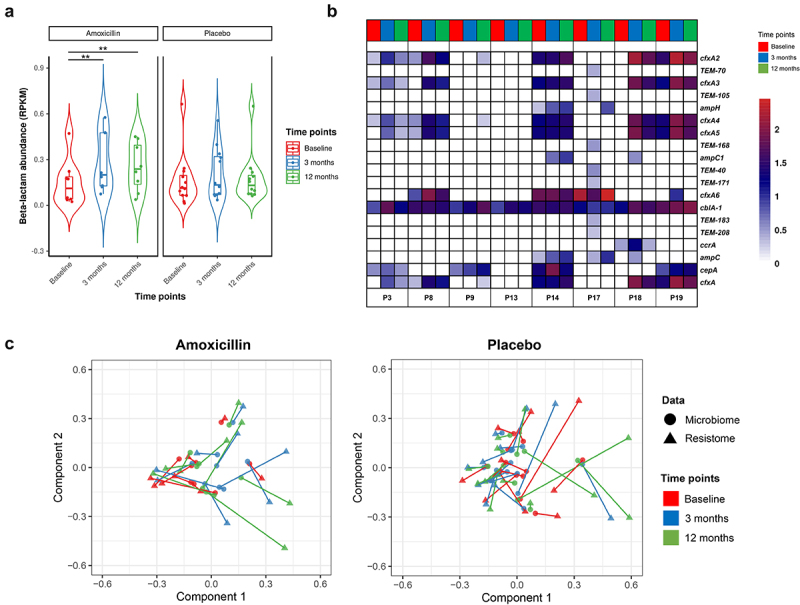
Impact of amoxicillin on abundance of beta-lactam ARGs and association between microbiome and resistome compositions. (a) Violin plots showing the relative abundance of class Beta-lactam measured as log10 of reads per kilobase million (RPKM) of Beta-lactam ARGs, stratified by treatment group and then time points. The changes in abundance were statistically evaluated using one-way ANOVA with repeated measures followed by post hoc pairwise t-test. The horizontal box lines represent the first quartile, the median and the third quartile. Whiskers denote the range of points within the first quartile − 1.5× the interquartile and the third quartile + 1.5× the interquartile. (Adjusted *P* values (*P*): ***P < .001, **P < .01; *P < .05 one-way ANOVA with repeated measures followed by post-hoc pairwise t-test). (b) Heat map displaying the log-transformed RPKM relative abundance of beta-lactam ARGs identified in amoxicillin-exposed patients. The x-axis displays all the samples at different time points which are stratified by the participants. (c) Procrustes analysis of resistome composition (filled triangles) and species composition (filled circles) for amoxicillin (left) and placebo (right)-treated groups using Hellinger transformation and PCoA ordination. The points are colored based on sampling time points in both groups. The length of line connecting two points indicates the degree of dissimilarity or distance between microbiome and resistome composition of the same sample.

Additional investigation of AMR determinants into the gut resistome revealed a common targeted response toward the amoxicillin treatment at the ARG-Class level. The class which directly corresponded to the treatment, i.e., beta-lactam, significantly increased in abundance in the amoxicillin group. Such effect persisted even after 9 months completion of the treatment (LEfSe) (Figure 5a). Also, the mean relative abundance of multidrug class increased by more than fourfold (log_2_ FC > 2) compared to baseline after amoxicillin treatment, while several other ARG classes with a threefold increase in relative abundances were observed over time in both the amoxicillin and placebo groups. Thus, such changes could not be linked to antibiotic treatment (Supplementary Figure 6). At the gene level, there was a high inter-individual variation of increase in abundances or emergence of beta-lactamase ARGs noticed after amoxicillin intervention (Figure 5b). The most consistent effect of the antibiotic was a threefold increase in abundance of *cfxA* beta-lactamase and its gene variants (*cfxA2, cfxA3, cfxA4 and cfxA6*) at 3 months in five out of eight amoxicillin-exposed participants. The abundance of these genes remained higher at 9 months post-treatment compared to their baseline levels. These *cfxA* beta-lactamase family resistance genes are commonly found in multiple bacterial genera, including Bacteroides, and are associated with penicillin and cephamycin resistance. Notably, the relative abundance of Bacteroides species was significantly increased in the amoxicillin patients, indicating that Bacteroides may have harbored the *cfxA* genes. Similarly, another clinically relevant beta-lactamase ARG, i.e., *cepA* encoding resistance to cephalosporins enriched immediately following antibiotic intake in patients, when present above detection levels at baseline (four out of eight patients). Additionally, we observed a post-antibiotic increase in abundances or induction of *ampC*-type beta-lactamase ARGs (*ampC, ampC1* and *ampH*) in three patients. Interestingly, their relative abundance pattern is congruent with *Escherichia coli*, a known microbial host harboring these ARGs. Further, we detected several of the TEM beta-lactamase ARGs (*TEM-70, TEM-168, TEM-105, TEM-171, TEM-183, TEM-205*) in one participant (P17) that were initially undetectable and selected toward higher abundance immediately after antibiotic exposure (3 months) and returned to lower levels following antibiotic withdrawal. These TEM-genes are potentially clinically relevant ARGs because they confer resistance toward commonly used antibiotics, including penicillins and cephalosporins, and because they are known to be precursors for beta-lactamases with an extended spectrum of resistance (ESBLs). Also, a fivefold increase in abundance of another clinically significant metallo beta-lactamase ARG (*ccrA*) encoding resistance to last-resort antibiotic (i.e., carbapenem) was observed for patient P18 at 3 months (Figure 5b).

Finally, the Procrustes analysis revealed that the resistome composition (ARG level) was significantly correlated with the microbiome (species level) in both placebo (Protest: sum of squares (m^2^) = 0.21; *R*^2^ = 0.88; *P* = .001; permutations = 999) and amoxicillin (Protest: sum of squares (m^2^) = 0.27; *R*^2^ = 0.85; *P* = .001; permutations = 999) groups, indicating that microbial community composition shapes the ARG distribution in the human gut. However, the overall correlation was more robust in placebo. More importantly, the baseline has a tighter correlation than post-treatment samples in the amoxicillin group, supporting the hypothesis that additional factors, such as antibiotic exposure, may have influenced the resistome changes independently of the microbiome (Figure 5c & Supplementary Table S5).

### Functional characterization of the antibiotic resistance reservoir in human gut microbiota

To functionally investigate the resistome, including the diversity of ARGs that remain undetected by conventional AMR databases in the gut microbiome, we next complemented shotgun metagenomics with functional metagenomic analysis. The constructed functional libraries encompassed a total of ~14.7 gigabase (Gb) pairs of metagenomic DNA with library sizes of 6Gb (baseline), 4.4Gb (3 months), and 4.3Gb (12 months), respectively, and an average insert size of 1.5–kb. Resistance was identified against 8 out of the 14 antibiotics screened. Importantly, we did not recover any resistant transformants for all the last-resort antibiotics tested, including meropenem, colistin and tigecycline. These antibiotics are considered as the last line of defense against infection caused by multidrug-resistant organisms (MDROs). In addition, none of the libraries constructed from feces of antibiotic-treated adult individuals retrieved ciprofloxacin, cefoxitin or gentamicin resistance (Supplementary Figure 7). Of the 42 selections performed, 24 yielded antibiotic-resistant *E. coli* transformants, of which 19 resulted in successfully sequenced and assembled libraries. On average, we assembled 3082 contigs with lengths greater than 500 bp with 4083 ORFs predicted in these contigs (Supplementary Table S2). In total, 1089 complete sequences were assigned to antibiotic resistance functions after hierarchical annotation of ORFs using multiple AMR databases, representing 599 unique sequences. These functionally discovered ARGs included multiple beta-lactamase classes (mainly class A and no class C), transcriptional regulators, multidrug efflux pumps, transporter proteins, acetyltransferases, aminotransferases, resistance modulators, aminoglycoside-modifying enzyme, tetracycline resistance protein, among others. Most of the functional ARGs (89.31%) were annotated through the Resfams-full database that contains additional HMM profiles of specific AMR genes that can contribute to antibiotic resistance, such as acetyltransferases, AraC transcriptional regulators, and ATP-binding cassette (ABC) efflux pumps.

**Figure 6.**
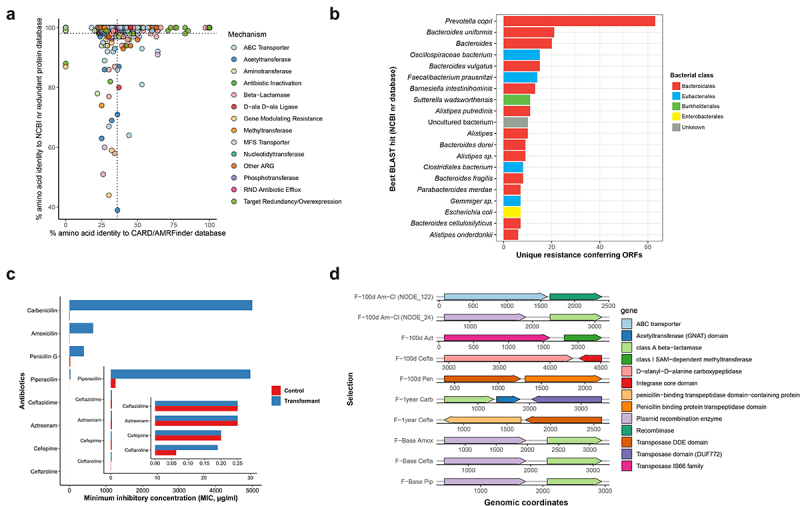
Functional metagenomic screening using *E. coli* as a surrogate host reveals an enriched gut resistome in amoxicillin exposed patients. (a) Amino acid identity between all of the functionally identified ARGs and their top hit in CARD/AMRFinder versus their top hit in the NCBI non-redundant protein database, colored by their mechanism of action. The vertical and horizontal dotted lines indicate the mean percentage identity of functional ARGs in the CARD/AMRFinder and NCBI database, respectively. (b) Horizontal bar plot showing the top 20 most commonly predicted hosts of functionally selected ARGs on the basis of maximum identity BLAST hits in the NCBI non-redundant protein database. Different colored bars represent different bacterial species orders. (c) Minimum inhibitory concentrations (MICs) of putatively novel ARG for *Escherichia coli* pZE21 transformants producing class A beta-lactamase and *Escherichia coli* pZE21 without the insert. This novel ARG has 100% amino acid identity to serine hydrolase (NCBI GenBank) and 38% of amino acid identity to known class A beta-lactamase (CARB-11) gene identified using BLASTP against the Comprehensive Antibiotic Resistance Database (CARD). Bars represent the average value from three independent experiments each. (d) Synteny of functionally selected ARGs with mobile genetic elements. Putative mobile genetic elements were identified syntenic to ARGs recovered from amoxicillin-clavulanate (a,b), aztreonam (c), ceftazidime (d, g, i), penicillin G (e), carbenicillin (f), amoxicillin (h) and piperacillin (j) selections. The x-axis shows the metagenomic coordinates.

The mean identity of these functionally selected ARGs to the CARD and AMRFinder-Protein database was 35.8% (median: 33%), whereas their mean identity to NCBI non-redundant protein entries was 98.1% (median 100%) (Figure 6a). This indicates that while most of the functionally identified ARGs in our selections are similar to previously sequenced proteins, these ARGs are remarkably infrequent in the current AMR databases. Interestingly, we also found that several of the functionally identified genes have already been annotated as ARGs in the NCBI database but are not functionally characterized with similar approach (HMM-based), across various habitats and environments in large-scale studies.^51,53,57^ The predicted microbial hosts of these functional ARGs (determined by the best BLAST hit to the NCBI non-redundant protein database) were predominantly commensal bacteria from the order Bacteroidales (Figure 6b). The detection of these commensal Bacteroidales as the potential hosts of ARGs in the human gut microbiome is not surprising and is in concordance with the current understanding of Bacteroidales as traffickers and prolific hosts of ARGs.^12,58–60^ Additionally, we identified uncultured bacterium as one of the top potential hosts of these ARGs (11 ORFs), suggesting that even not well-represented uncultured organisms can be important sources of ARGs in the gut microbiome.

We next investigated the influence of amoxicillin intake on the prevalence of functionally discovered ARGs over time. To avoid false positives and over-inflation of ARGs abundance levels in the sequenced metagenomes, we only mapped ARGs identified using the Resfams-core database to the shotgun data.^42,61^ We noticed a similar pattern as for the shotgun metagenomics of high inter-individual variation in the abundance of functional ARGs in the gut microbiome of the treated subjects (Supplementary Figure 8A). These ARGs were found both in the treated and in the placebo subjects. The abundance of functional beta-lactamases slightly increased after treatment in the amoxicillin-treated subjects, though, unlike shotgun metagenomic data, these changes were not statistically significant (Supplementary Figure 8B). In comparison, no such trend was noticed in the placebo group.

We also discovered at least 13 putatively novel ARGs in the gut microbiome of antibiotic-treated patients. No sequence homologs were detected for eight of these ARGs in known AMR sequence databases. The list of putatively novel ARGs annotated based on AMR (CARD, AMRFinder) and NCBI non-redundant protein database is presented in Table 2. We further experimentally validated a putative class A beta-lactamase resistance protein with 100% amino acid identity to serine hydrolase and 38% identity to known beta-lactamase resistance gene (CARB-11) by expressing it in *E. coli*. The results showed that this novel ARG conferred resistance toward carbenicillin, as observed by the increase in the minimal inhibitory concentration (MIC) by more than 400-fold compared to the control. For amoxicillin, we found approximately a 108-fold (650 μg/ml) increase in the MIC values. It also demonstrated reduced susceptibility, ranging from 16- to 30-fold, to other penicillins such as penicillin G and piperacillin. These are typical characteristics of class A carbenicillin-hydrolyzing beta-lactamases (CARB-type) (Figure 6c and Additional file S4).

**Table 2.** Putative novel functional ARGs.

ORF Info					CARD+AMRFINDER Database			NCBI Nr Protein Database				Global alignment of best top local hit			
Name	Selection	Length	Source	Protein type	Protein ID	Identity (%)	Coverage (%)	Description	Protein ID	Identity (%)	Coverage (%)	Description	Identity (%)	Note	
NODE_23_length_2861_cov_41.484676_2_#_339_#_1250_#_1_#_ID = 23_2;partial = 00;start_type = ATG;rbs_motif = None;rbs_spacer = None;gc_cont = 0.569,NODE_34_length_2830_cov_744.602162_2_#_338_#_1249_#_1_#_ID = 34_2;partial = 00;start_type = ATG;rbs_motif = None;rbs_spacer = None;gc_cont = 0.569	F-100d Pip, F-100d Amox	303	ResFams-core	Class A beta-lactamase	WP_063857838.1	38	82	CARB-11	HJA09995.1	99.01	100	serine hydrolase	99	Functional validated	
NODE_17_length_2193_cov_247.850327_2_#_125_#_2065_#_1_#_ID = 17_2;partial = 00;start_type = ATG;rbs_motif = None;rbs_spacer = None;gc_cont = 0.567	F-1 year Cefta	646	Resfams-full	Penicillin binding protein transpeptidase domain	WP_000138348.1	31	84	pbp2	OLA80974.1	99.36	96	hypothetical protein BHW58_06090 [Azospirillum sp. 51_20] (624)	96		
NODE_524_length_1351_cov_123.915895_2_#_247_#_1158_#_-1_#_ID = 524_2;partial = 00;start_type = ATG;rbs_motif = GGAG/GAGG;rbs_spacer = 5–10bp;gc_cont = 0.622	F-100d Pip	303	Resfams-full	Beta-lactamase superfamily domain	AWN09461.1	22	65	metallo-beta-lactamase PNGM-1	WP_092366234.1	100	100	ribonuclease Z	100		
NODE_17_length_3055_cov_210.484000_2_#_552_#_1472_#_1_#_ID = 17_2;partial = 00;start_type = TTG;rbs_motif = 4Base/6BMM;rbs_spacer = 13–15bp;gc_cont = 0.450	F-1 year Am-Cl	306	Resfams-full	Beta-lactamase superfamily domain	AWN09461.1	24	45	metallo-beta-lactamase PNGM-1	WP_206813282.1	100	99	ribonuclease Z	99.3		
NODE_3401_length_519_cov_1.668103_2_#_72_#_509_#_1_#_ID = 3401_2;partial = 00;start_type = ATG;rbs_motif = None;rbs_spacer = None;gc_cont = 0.562	F-100d Am-Cl	145	Resfams-full	Acetyltransferase (GNAT) domain	WP_046928126.1	31	80	aacA	OLA82746.1	99	98.61	hypothetical protein BHW58_00500 [Azospirillum sp. 51_20]	77.2		
NODE_295_length_1136_cov_5.179463_1_#_86_#_979_#_1_#_ID = 295_1;partial = 00;start_type = ATG;rbs_motif = None;rbs_spacer = None;gc_cont = 0.511	F-Base Pen	297	Resfams-full	Aminotransferase class I and II	No hit	-		-	WP_006846964.1	100	100	pyridoxal phosphate-dependent aminotransferase	73.7		
NODE_336_length_1177_cov_8.886809_1_#_22_#_528_#_-1_#_ID = 336_1;partial = 00;start_type = ATG;rbs_motif = TATAA;rbs_spacer = 3bp;gc_cont = 0.491	F-1 year Pen	168	Resfams-full	Aminotransferase class I and II	No hit	-		-	GKH14396.1	100	89	hypothetical protein CE91St12_26060	84		
NODE_97_length_1542_cov_21.211163_2_#_292_#_1443_#_-1_#_ID = 97_2;partial = 00;start_type = ATG;rbs_motif = AAAAA;rbs_spacer = 8bp;gc_cont = 0.443	F-Base Pip	383	Resfams-full	Aminotransferase class I and II	No hit	-		-	ALK85074.1	100	100	8-amino-7-oxononanoate synthase	97.7		
NODE_35_length_2999_cov_27.911685_1_#_292_#_1488_#_1_#_ID = 35_1;partial = 00;start_type = ATG;rbs_motif = None;rbs_spacer = None;gc_cont = 0.596	F-100d Am-Cl	398	Resfams-full	Aminotransferase class I and II	No hit	-		-	WP_014775721.1	100	100	pyridoxal phosphate-dependent aminotransferase	100		
NODE_88_length_1596_cov_2.811811_2_#_162_#_1244_#_-1_#_ID = 88_2;partial = 00;start_type = ATG;rbs_motif = None;rbs_spacer = None;gc_cont = 0.494	F-100d Cefta	360	Resfams-full	Aminotransferase class I and II	No hit	-		-	MBN2914949.1	99	100	PatB family C-S lyase	90.2		
NODE_90_length_1589_cov_8.839635_2_#_275_#_1294_#_1_#_ID = 90_2;partial = 00;start_type = ATG;rbs_motif = TAA;rbs_spacer = 11bp;gc_cont = 0.496	F-100d Cefta	339	Resfams-full	Aminotransferase class IV	No hit	-		-	WP_005833410.1	100	100	branched-chain amino acid aminotransferase	100		
NODE_59_length_2694_cov_208.519515_3_#_1905_#_2483_#_-1_#_ID = 59_3;partial = 00;start_type = ATG;rbs_motif = TAAA;rbs_spacer = 6bp;gc_cont = 0.446	F-100d Amox	192	Resfams-full	Acetyltransferase (GNAT) domain	No hit	-		-	WP_005784400.1	100	100	GNAT family N-acetyltransferase	100		
NODE_8_length_3480_cov_21.774015_2_#_373_#_2712_#_1_#_ID = 8_2;partial = 00;start_type = ATG;rbs_motif = AGGAGG;rbs_spacer = 5–10bp;gc_cont = 0.554,NODE_3_length_3481_cov_12.052248_2_#_769_#_3108_#_-1_#_ID = 3_2;partial = 00;start_type = ATG;rbs_motif = AGGAGG;rbs_spacer = 5–10bp;gc_cont = 0.555	F-100d Am-Cl,F-100d Amox	779	Resfams-full	ABC-2 type transporter	No hit	-		-	WP_119107293.1	100	100	YhgE/Pip domain-containing protein	99.6		

To underline the potential of horizontal ARG transfer within the gut microbiome, we explored the presence of mobile genetic elements in the assembled contigs (in synteny with ARGs). 4.43% of the total contigs encoded a putative mobile genetic element. These were observed in all selections against beta-lactam antibiotics, commonly appearing in penicillin, amoxicillin and piperacillin selections, but also in lower numbers in, the ceftazidime selections (Supplementary Figure 9B). Nevertheless, antibiotic treatment differences did appear. We noticed a significantly higher load of putative MGEs in post-antibiotic treatment selections than in baseline selections in our functional metagenomic data (Supplementary Figure 9A) (pairwise Wilcoxon test). Interestingly, these post-treatment selections had the most putative MGE-associated annotations despite lower sequencing reads and lower input libraries. MGEs colocalized with AMR genes are of serious concern due to the ease of their spread. In particular, we recovered a functionally identified class A beta-lactamase ARG colocalized with a plasmid element selected in penicillins (amoxicillin, piperacillin, amoxicillin-clavulanate) and cephalosporin (ceftazidime). Such ARGs with typical characteristics of extended-spectrum beta-lactamases (ESBLs) can play a crucial role in resistance dissemination (Figure 6d). All these findings emphasize the usefulness of functional metagenomics as a culture-independent and sequence-unbiased approach for resistome characterization.

## Discussion

In this study, we explored the ecological side effects of prolonged amoxicillin exposure on the human gut microbiome and resistome. We found that long-term amoxicillin therapy in a relatively healthy population had more pronounced yet transient effects on microbiome composition. This was in contrast with the effects on the resistome, for which changes persisted for long-term after treatment cessation. Enrichment of antibiotic resistance genes was mostly specific to beta-lactam antibiotics, including instances showing enrichment of beta-lactamase genes associated with extended spectrum of activity (ESBL) and with action against carbapenems.

Perturbations in gut microbiome balance by short-course amoxicillin interventions have reported minor or not significant effects on microbiome composition.^30–32,62^ Our results indicate that in contrast to short-course, prolonged use of amoxicillin can have severe side-effects. We found that amoxicillin had a common and reproducible impact on the microbiome with most noticeable changes observed in the species with lower abundance. Among these were known short-chain fatty acid (particularly butyrate) producers, significantly depleted upon antibiotic therapy completion. Contrarily, bacterial genera *Bacteroides*, well-known for carrying resistance to beta-lactams, systematically increased upon amoxicillin treatment.^60,63^ In addition, we validated previous findings where the potential marker of beta-lactam or antibiotic-induced dysbiosis, i.e., *L. bolteae*, significantly enriched upon amoxicillin treatment.^18,64^ Upon treatment, we also observed depletion of a recently discovered health-associated species, i.e., *Oscillibacter* sp. *57_20*.^56^ The effects on microbiome composition were short-lived as the microbiome returned to pre-treatment levels at 9 months after amoxicillin treatment, which can be explained by community resilience.^33,65^ Despite their transience, such microbial disturbances can also have negative health-related consequences such as increased susceptibility to recurrent *Clostridioides difficile* infections, which can be more detrimental for patients with an already dysbiotic microbiome (diseased).^21,66^

Further, our work also highlights that amoxicillin exposure led to a short-term but significant increase in microbiome dissimilarity between baseline and post-treatment samples. The possible explanation behind such diversification would be considerable variations in baseline microbiome composition across patients, which may result in different levels of severity and vulnerability to antibiotic perturbation. The influence of inter-individual variability on microbiome responses was particularly evident on the resistome results, with a higher inter-individual variability in response to amoxicillin for the resistome than the microbiome. Such difference may reflect the fact that most of the ARGs are specific to individuals, while microbial taxa are more conserved across patients. In line with our results, individualized responses to antibiotic interventions have also been reported for resistome studies evaluating short-course antibiotic therapies.^18,67,68^ Notably, no significant changes were observed in the microbiome and resistome diversity and composition over time in patients who did not take amoxicillin, thus confirming the stability, resilience and robustness of the healthy, adult human gut microbiome.^69,70^

Complementation of whole metagenomic sequencing with functional metagenomics enables broader characterization of resistance determinants in metagenomes.^42,45^ Functional metagenomics provides not only functional information on the ARGs present in microbial communities but also enables the discovery of novel ARGs. The technique utilizes a heterologous host, e.g. *E. coli*, for gene expression via library cloning which poses as a strong advantage given that it allows for the identification of ARGs from uncultured bacteria. We found that one of the top potential hosts of functionally identified ARGs included an uncultured bacterium, along with the well-known and predominant (identified through shotgun metagenomics) Bacteroides and Prevotella species. Notably, although we did not identify resistance against last resort antibiotics in the functional screening, some were detected by whole metagenomic sequencing, thus highlighting the complementarity of the two methods. We have also discovered several putatively novel ARGs (1 validated functionally) in the gut microbiome of the amoxicillin-treated patients, with most of them detected in selections from post-treatment libraries. Lastly, detection of MGEs mediating the spread of ARGs (colocalized) in our functional metagenomic data highlights the likelihood of a mobilizable resistome within the human gut microbiome. Interestingly, most putative MGE-associated annotations were in the post-treatment samples, suggesting an additional possible collateral effect of prolonged amoxicillin therapy.

The small treatment group size limits the generalization of our conclusions and makes it difficult to discern significant relationships at the population level, mainly due to high inter-individual variability and personalized response to antibiotics perceived in our data. Nonetheless, we rigorously controlled for such variability by using individuals as their controls in pre- and post-treatment comparisons. Moreover, additional sampling time points before and during antibiotic administration, would have provided a better understanding of the dynamics and natural variability of the human gut microbiome overtime. Functional metagenomics was employed to complement the resistome information from whole metagenomic analysis. While it provides precious information based on phenotypic characterization, this approach is still limited in scope due to definite classes of antibiotics and the lack of efficient cloning systems for expression in Gram-positives.^71^ Additionally, the isolation of mobile genetic elements colocalized with ARGs is technically constrained, as little information regarding the genetic background of the functional ARGs is available because of small DNA fragments (insert) size in our functional selections. Still, functional metagenomics is the only high-throughput metagenomics method that enables identification of completely novel ARGs. Future multicenter, longitudinal sequencing-based studies with increased sampling density, complemented with functional metagenomics assays using multi-host-systems would have the potential to provide solid evidence of relevance for antibiotic stewardship practices. However, large cohort prospective studies to investigate the impact of prolonged use of antibiotics in humans would need special ethical considerations regarding benefits and risks, which only in rare cases would be justifiable. Such considerations highlight the critical relevance of the high-throughput microbiome and resistome analysis of unique data from our randomized trial of relatively healthy individuals, despite limitations in sample size.

In conclusion, our results provide compelling evidence that the human adult gut microbiome and resistome respond differently to prolonged amoxicillin treatment. The persistent increase in AMR abundance and diversity along with enrichment of beta-lactam resistance genes long after antibiotic use termination, including resistance genes to last resort antibiotics detected by whole metagenomic sequencing, highlights the risks associated with prolonged antibiotic exposure. Such risks need to be considered in view of potential benefits. Patients with chronic low back pain and Modic changes included in the current study were part of a multicenter study in Norway that showed a lack of significant effects of prolonged amoxicillin use in self-reported measures of disability, pain intensity or quality of life.^35^ Thus, our study adds an additional layer of support against the general recommendation of antibiotic therapy for chronic low back pain and Modic changes and emphasizes the importance of considering such risks in relation to other conditions with no or minor benefits from prolonged antibiotic therapy.

## Supplementary Material

Supplemental Material

## Data Availability

Clean shotgun metagenomic reads after removal of human DNA and assembled functional metagenomic contigs are available at NCBI SRA under BioProject ID: PRJNA894204. Additional participants metadata (deidentified) will be available upon request addressed to kjersti.storheim@medisin.uio.no, in accordance with local registration and ethical approval.
