# Supplementary material for: Differential response to prolonged amoxicillin treatment: long-term resilience of the microbiome versus long-lasting perturbations in the gut resistome
Source: Gut Microbes. 2022 Dec 28;15(1):2157200. doi: 10.1080/19490976.2022.2157200 (PMC9809947; doi:10.1080/19490976.2022.2157200)
Supplement: Supplemental Material [file KGMI_A_2157200_SM8536.zip › Supplementary information/File S4 Validation of the novel class A beta-lactamase gene.docx]

# Functional MODIC validation

1. **Extraction of the plasmid from the libraries F-100d Amox.**

Plasmids from the libraries were extracted using the ZymoPure™ Plasmid Miniprep Kit (Zymo Research) following the manufacturer’s instructions.

# Amplification of the novel gene with PCR:

Per 50µl PCR reaction

- 5X Phusion HF Buffer10 µL
- 10 mM dNTPs 1 µL
- 10 µM Forward Primer FP2313 (see Appendix): 0.3 µL
- 10 µM Reverse Primer FP2314 (see Appendix): 0.3 µL
- Phusion DNA Polymerase: 0.5 µL
- Template DNA: 0.3µl
- Nuclease-free water: 37.6µl

PCR cycling conditions

- 98°C for 10 minutes
- 98°C for 10 seconds
- 64°C for 30 seconds
- 72°C for 40 seconds
- Go to the second step; repeat 38 times
- 72°C for ten minutes
- 4°C indefinitely

The PCR product was purified using DNA Clean & Concentration®-5 kit and subjected to gel electrophoresis (1% agarose, 0.5x Tris-Borate-EDTA (TBE), SYBR™ Safe (Invitrogen)) at 65V for 35 minutes. From this, the band (912bp) was excised using MinElute® Gel Extraction Kit according to the manufacturer's protocol.

# Plasmid extraction and linearization

*E. coli* with *pZE21* MCS-1 expression vector were grown overnight in LB with (50µg/mL kanamycin) at 600 RPM and 37°C. Colonies were collected by centrifugation at 10000 x g for 8 min before extraction of the plasmids using GeneJET plasmid Miniprep kit (Thermo Fisher) according to the manufacturer protocol.

PCR was performed as follows:

|  | **1X** | **4X** |
| --- | --- | --- |
| Circular *pZE21 MCS-1* | 25µl | 100µl |
| Forward primer FP1984 (See Appendix) | 0.3µl | 1.2µl |
| Reverse primer FP1985 (See Appendix) | 0.3µl | 1.2µl |
| 5X Buffer Q5 | 10µl | 40µl |
| dNTP Stock 10mM | 1µl | 4µl |
| Q5 DNA polymerase | 0,5µl | 2µl |
| Nuclease free water | 12.9µl | 51.6µl |
| **Total** | **50µl** | **200µl** |

PCR cycling conditions

- 98°C for 30 seconds
- 98°C for 10 seconds
- 65°C for 30 seconds
- 72°C for 30 seconds
- Go to the second step; repeat 30 times
- 72°C for two minutes
- 4°C indefinitely

The four PCR reactions were Collected in one Eppendorf tubes, purified using DNA Clean & Concentration®-25 kit (Zymo Research), before running the gel electrophoresis (1% agarose, 0.5x Tris-Borate-EDTA (TBE), SYBR™ Safe (Invitrogen)) at 65V for 60 minutes. Following that, the band (~2200bp) was excised using QIAquick® Gel Extraction Kit (Qiagen) according to the manufacturer's protocol.

# Blunting and phosphorylation of DNA

The END-It™ DNA End Repair kit (Lucigen) was used to end repair the DNA: For each 34 μL volume of a size-selected gene, add the following:

- - 5 μL 10× End-Repair Buffer
  - 5 μL dNTP mix (2.5 mM)
  - 5 μL ATP (10 mM)
  - 1 μL End-Repair Enzyme Mix
  - Incubate at room temperature for 45 min.
  - Heat-inactivate the reaction at 70 °C for 10 min.
  - Purify DNA using Zymo DNA Clean & Concentration®-5 kit (Zymo Research).
  - Elute the DNA with 12 μL of nuclease-free water.

# Dephosphorylation of the plasmid

Dephosphorylate purified plasmid using Antarctic phosphatase (NEB):

- 16µl purified plasmid (1µg)
- 2µl Antarctic phosphatase reaction buffer
- 1µl Antarctic phosphatase
- 1µl nuclease-free water
- Incubate at 37^o^C for 1 hour.
- Inactivate at 80^o^C 2 min.
- Purify plasmid using Zymo DNA Clean & Concentration®-5 kit (Zymo Research).
- Elute in 12µl nuclease-free water.

# Ligation and Dialysis

Ligation reaction using end-repaired gene and linearized vector was performed using the Fast-Link™ DNA Ligation Kit.

5:1 molar ratio of insert: vector for ligation was maintained.

A 15 μL reaction tube with the following reagents was prepared as follows:

- Insert (9µl)
- Plasmid (0.8µl)
- 1.5 μL Fast-Link 10× Ligation buffer
- 0.75 μL 10 mM ATP
- 1 μL Fast-Link DNA ligase (2 U/ μL)
- Nuclease free water to a total volume of 15 µl (1.95 µl)
- Incubate the reaction at room temperature overnight.
- Heat-inactivate reaction by incubating for 15 min at 70 °C.

After heat inactivation, the ligation reaction was dialyzed by filling a clean petri dish with 20 mL of nuclease-free water, place a 0.025 μm nitrocellulose membrane (Merck ref. VSWP02500) on top of the water so that it floats. After that, the entire volume of the ligated product was transferred carefully to the membrane before closing the lid, incubating for 45 min, and collecting the sample in a clean 1.5 mL tube.

# Electroporation

A gap sterile electroporation cuvette (0.1cm), 1.5ml tube, ligated DNA, and electrocompetent cells (Lucigen E. cloni 10G, ref. 60081-2) were placed on ice. Next, electrocompetent cells (25 μl) were aliquoted to a prechilled tube on ice. Then, the entire ligation reaction volume (~15 μL) of the sample was added to the aliquoted electrocompetent cells. The same was done with the negative control. Next, electroporation was perform using 0.1 cm cuvette with μF, 600 Ω, and 1800 V, and the cells and recovery medium were transferred to clean 2ml tubes, before shacking on a thermomixer for 1 hour, 500 RPM, 37 °C.

From this, three LB (250mL) were prepared, and 2700µl and 125 µl of amoxicillin (10mg/mL) and kanamycin(100µg/mL), respectively in one of them which allocated for Amox/km plates, while only kanamycin for Km plates and only LB for control plates. PCR and gel electrophoresis were done for 5 chosen colonies on Amox/km plate as follows:

- 5X Phusion HF Buffer10 µL
- 10 mM dNTPs 1 µL
- 10 µM Forward Primer FP2313 (see Appendix): 0.3 µL
- 10 µM Reverse Primer FP2314 (see Appendix): 0.3 µL
- Phusion DNA Polymerase: 0.5 µL
- Template DNA: pipette tip with selected colony
- Nuclease-free water: 37.6µl PCR cycling conditions
- 98°C for 10 minutes
- 98°C for 10 seconds
- 64°C for 30 seconds
- 72°C for 40 seconds
- Go to the second step; repeat 38 times
- 72°C for ten minutes
- 4°C indefinitely

The two positive colonies (with ~912bp on the gel) with negative control were cultured on MH plates after resuspension and dilution (1:100, 5µL in 495 µL PBS). Following this, two PCR reactions and gel electrophoresis were done for 5 chosen colonies on Amox/km plate as follows:

- 5X Phusion HF Buffer10 µL
- 10 mM dNTPs 1 µL
- 10 µM Forward Primer (FP2313 see Appendix): 0.3 µL
- 10 µM Reverse Primer (FP2314 see Appendix): 0.3 µL
- Phusion DNA Polymerase: 0.5 µL
- Template DNA: pipette tip with selected colony
- Nuclease-free water: 37.6µl PCR cycling conditions
- 98°C for 10 minutes
- 98°C for 10 seconds
- 64°C for 30 seconds
- 72°C for 40 seconds
- Go to the second step; repeat 38 times
- 72°C for ten minutes
- 4°C indefinitely And
- Q5 Buffer10 µL
- 10 mM dNTPs 1 µL
- 10 µM Forward Primer (FP1986 see Appendix): 0.3 µL
- 10 µM Reverse Primer (FP1987 see Appendix): 0.3 µL
- Q5 DNA Polymerase: 0.5 µL
- Template DNA: pipette tip with selected colony
- Nuclease-free water: 37.6µl PCR cycling conditions
- 98°C for 30 seconds
- 98°C for 10 seconds
- 65°C for 30 seconds
- 72°C for 30 seconds
- Go to the second step; repeat 30 times
- 72°C for two minutes
- 4°C indefinitely

For the gel electrophoresis with the same mentioned parameters, while the primers FP2313-2314 gave the correct size of the insert (912bp), the primers FP1986-1987 gave ~300bp for negative colonies and

~1212 for the positive colonies.

# MIC estimation

The positive colonies were tested with different concentrations of amoxicillin on a 96 well plate.

- Cfu colony 1: 1 900 000 000
- Cfu colony 2: 1 700 000 000
- Cfu *pZE21* negative control: 500 000 000

Mueller Hinton culture (10 mL) was made for the colonies and *pZE21* each. For each well, the culture (180 µL) with kanamycin (50 µL/mL) was added, in addition to control wells without antibiotics.

Transformants were tested with carbenicillin (concentration range 6-5000µg/mL), amoxicillin (concentration range 6-1300µg/mL), penicillin G (concentration range 25-600µg/mL), piperacillin (concentration range 1-60µg/mL), aztreonam (concentration range 0.06-0.5µg/mL), ceftazidime (concentration range 0.06-1µg/mL), cefoxitin (concentration range 0.06-0.5µg/mL), and cefepime (concentration range 0.05-0.8µg/mL). Culture plates were incubated at 37C for 48 hours and growth rates were observed by (OD600) during the entire period. The control inhibited at (12.5 µg/mL for carbenicillin, 6 µg/mL for amoxicillin, 25 µg/mL for penicillin G, 1 µg/mL for piperacillin, 0.25µg/mL for aztreonam, 0.25 µg/mL for ceftazidime, 25 µg/mL for cefoxitin, and 0.2µg/mL for cefepime), while the transformant inhibited at (5000µg/mL for carbenicillin, 650µg/mL for amoxicillin, 400µg/mL for penicillin G, 30µg/mL for piperacillin, 0.25µg/mL for aztreonam, 0.25µg/mL for ceftazidime, 12.5µg/mL for cefoxitin, and 0.2µg/mL for cefepime. Each experiment was performed three times and the average was presented. The transformant was tested with a ceftaroline strip (from Biomerieux) through the E-test and incubated for 24 hr at 37°C. While the control inhibited 0.064µg/ml, the transformant inhibited at 0.19µg/ml.

**Results**

| **Antibiotic** | **Control** | **Transformant** |
| --- | --- | --- |
| carbenicillin | 12.5 μg/mL | 5000μg/mL |
| amoxicillin | 6 μg/mL | 650μg/mL |
| penicillin G | 25 μg/mL | 400μg/mL |
| piperacillin | 1 μg/mL | 30μg/mL |
| Aztreonam | 0.25μg/mL | 0.25μg/mL |
| Ceftazidime | 0.25 μg/mL | 0.25μg/mL |
| Cefoxitin | 25 μg/mL | 12.5μg/mL |
| Cefepime | 0.2μg/mL | 0.2μg/mL |
| ceftaroline strip | 0.064μg/ml | 0.19μg/ml |

After phenotypical testing, the discovered gene presented increased resistance towards amoxicillin by around 108 times (650μg/ml) compared to the control (6 μg/ml). This concentration is higher than the breakpoint registered at the European Committee on Antimicrobial Susceptibility Testing (EUCAST) against *Enterobacteriaceae*, *Enterococcus* spp., *Streptococcus pneumoniae, Pseudomonas* spp*. Staphylococcus* spp. and *Streptococcus* groups A, B, C, and G by approximately 93 times. If one of the microorganisms above harbored this gene, it would potentially resist the penicillin G and piperacillin at MICs larger than that reported by the EUCAST by around 300 times and 4 times, respectively (EUCAST, 2021). The MIC estimated for the carbenicillin was higher than that was detected at the control by almost 400 times (Figure 38). Besides, the estimated MIC of carbenicillin (5000μg/ml) was more prominent than that was reported against *Pseudomonas* spp. by around five times. Fortunately, the bacteria carrying this gene could be eradicated by aztreonam, ceftazidime, cefoxitin, cefepime, and ceftaroline, as their estimated MICs were lower than what was stated by the Clinical Lab Standards Institute (CLSI) and EUCAST, apart from some identified resistance to the fifth generation cephalosporin, namely, ceftaroline.

**Appendix**

# >The Novel gene nucleotide sequence

ATGAACCTTCGCTTTCCTCTTTCATCTGTCTGTTCGCTTGTCACAATCGGTCTGTTTGC GGTCTCCGCAGTGACAGGGTGTGCAACGCCGCAAAACAGCGTTTCTCCAAAGCTGTC GCAGCAGGACTCGCAGAACGCCATTGTCACCATTGAACAAAAGTACGACGCCGCCAT CGGCGTGTCCCTTCGTGACGCAGAGGGCAAGGTGCTTTTGGAGTGGCGCAGTCGCGA GCGCTTCCCACTTACCAGCACCGTTAAAGCTCTCGAATGCGCGCGCGTCTACGAACT GGGGCTCGAAAACCGCAGCGCTCCGATCAAAACCACGCCTGTCGTACCGCATTCCCC CGTCTACGGAACGGTAAATCCCGACACGAAGGTCACGTTGAAGGAAGCATGCCGCGC CGCTTTGTCGCAAAGCGACAACCGCGCCGCCAACTTCATCTTTGTGCACACCGGCGG ACCGAAGGCCTTAACCAAGTGGCTGCGGCAAAAGGGCGACAACACCACCCGCAGCG ATCGTCTGGAACCGGATTTGAATCTGTCCGGAAAGAATGAATACCGAGACACGACGA CACCGAGCAATGCGTCGCTCAATTGGCAACGATTCGATACTCAGCTGCCCAAATCGG CCCGCAGCCAATGGCTGGCGGATCTTGCCGCCAATCAGATGGCCGGCAATCTGTTTC GCTCGCGTCTACCCGAAGGCTGGACGCTTTTCGACCGTTCCGGAGCCGGATCGGACG AATTCTGTGCTACCCGCGCCAATCATGCGATCTTGGTCACGGACAAAGGCGCTCGTTA TTACGCTGCCGTGCATTTAAAGGCTCCGGCCGGAACTCCGATGGAAAAGCGCGACGC CATTTTGCAACAAGCCATTGAGGTCGTCTACGCTCATCTAAAAAGTCGGCTTTAA

# Primers:

| **Primers** | **Sequence** |
| --- | --- |
| Forward primer (FP2313) | 5’ATGAACCTTCGCTTTCCTCTTTC |
| Reverse primer (FP2314) | 5’TTAAAGCCGACTTTTTAGATGAGC |
| Forward primer (FP1984) | 5’GACGGTATCGATAAGCTTGAT |
| Reverse primer (FP1985) | 5’GACCTCGAGGGGGGG |
| Forward primer (FP1986) | 5’GATACTGAGCACATCAGCAGGA |
| Reverse primer (FP1987) | 5’CCTGATTCTGTGGATAACCGTA |
