# Supplementary material for: Differential response to prolonged amoxicillin treatment: long-term resilience of the microbiome versus long-lasting perturbations in the gut resistome
Source: Gut Microbes. 2022 Dec 28;15(1):2157200. doi: 10.1080/19490976.2022.2157200 (PMC9809947; doi:10.1080/19490976.2022.2157200)
Supplement: Supplemental Material [file KGMI_A_2157200_SM8536.zip › Supplementary information/Table S5 Procrustes analysis result.docx]

| **Table S5 Procrustes analysis result statistics** |  |  |  |
| --- | --- | --- | --- |
| **1. Time points** |  |  |  |
|  | **Baseline** | **3 months** | **12 months** |
| Procrustes Sum of Squares (m12 squared) | 0.2724 | 0.3341 | 0.2944 |
| Correlation in a symmetric Procrustes rotation | 0.853 | 0.816 | 0.84 |
| Significance | 0.001 | 0.034 | 0.003 |
| Permutation | free | free | free |
| Number of permutations | 999 | 999 | 999 |
|  |  |  |  |
| **2. Treatment Group** |  |  |  |
|  | **Amoxicillin** | **Placebo** |  |
| Procrustes Sum of Squares (m12 squared) | 0.2721 | 0.2132 |  |
| Correlation in a symmetric Procrustes rotation | 0.8532 | 0.887 |  |
| Significance | 0.001 | 0.001 |  |
| Permutation | free | free |  |
| Number of permutations | 999 | 999 |  |
