# Supplementary figures and images for: Differential response to prolonged amoxicillin treatment: long-term resilience of the microbiome versus long-lasting perturbations in the gut resistome
Source: Gut Microbes. 2022 Dec 28;15(1):2157200. doi: 10.1080/19490976.2022.2157200 (PMC9809947; doi:10.1080/19490976.2022.2157200)

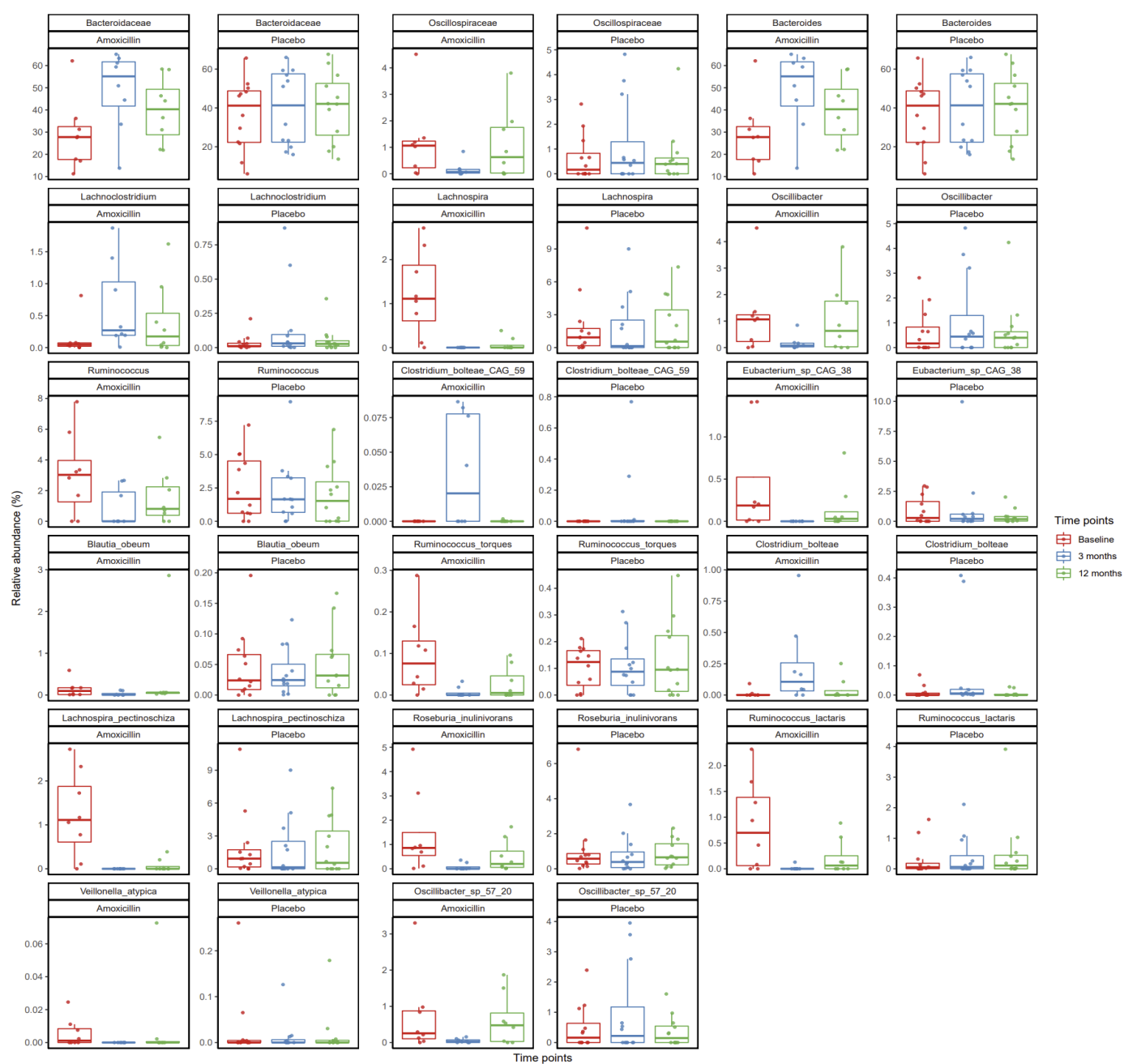

Supplementary Figure 1

Supplement: Supplemental Material [file KGMI_A_2157200_SM8536.zip › Supplementary information/Supplementary Figure 1.pdf]

**A**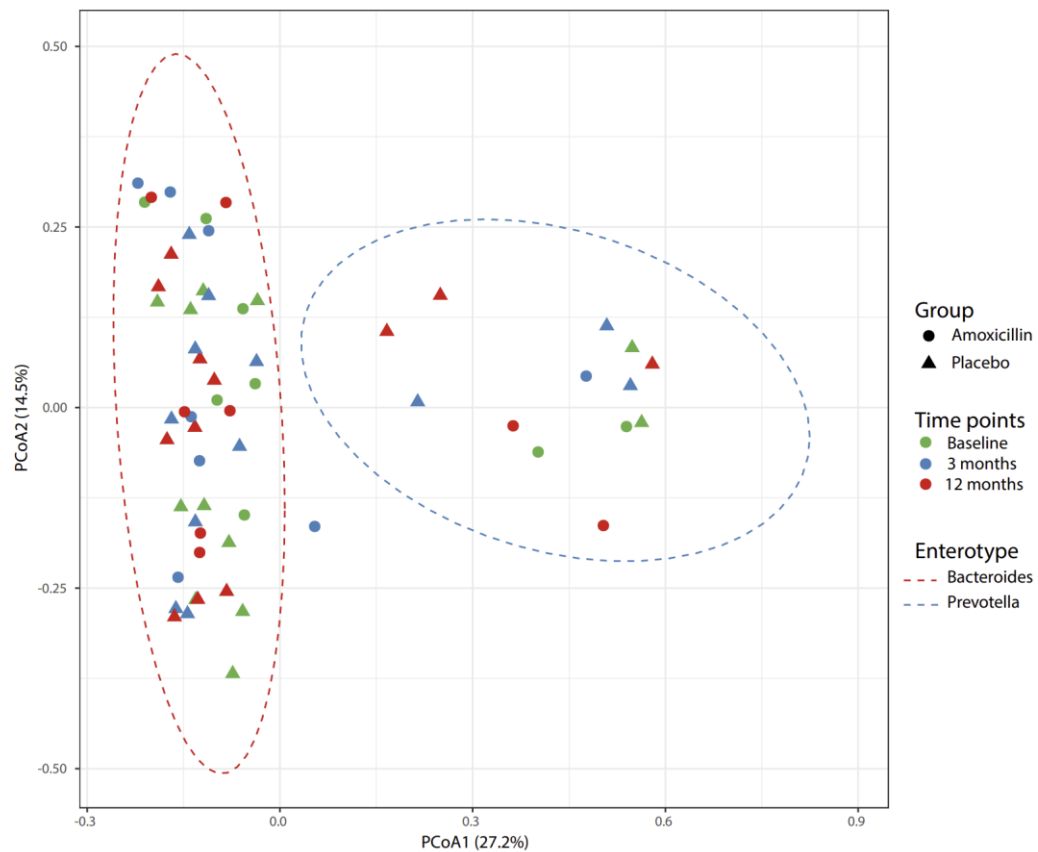**B**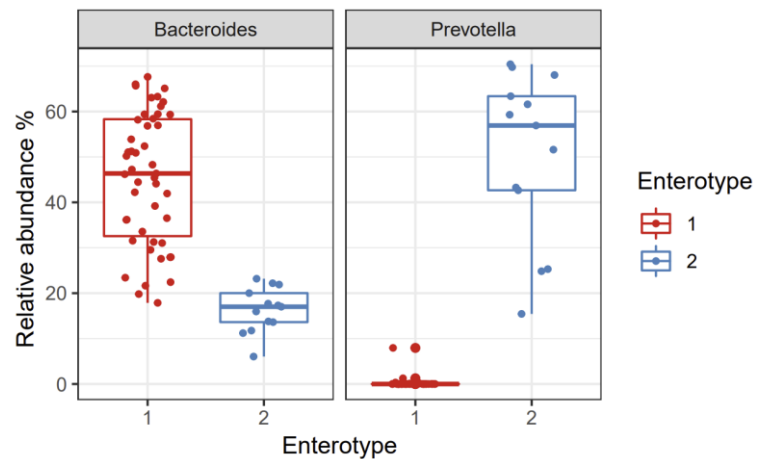

**Supplementary Figure 2**

Supplement: Supplemental Material [file KGMI_A_2157200_SM8536.zip › Supplementary information/Supplementary Figure 2 new.pdf]

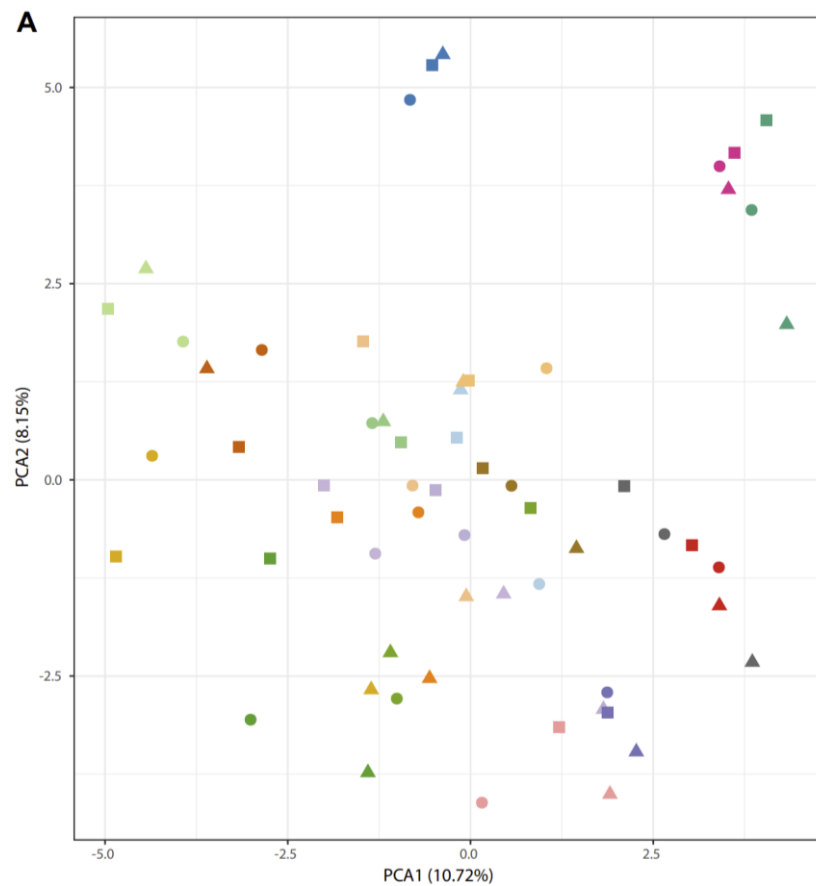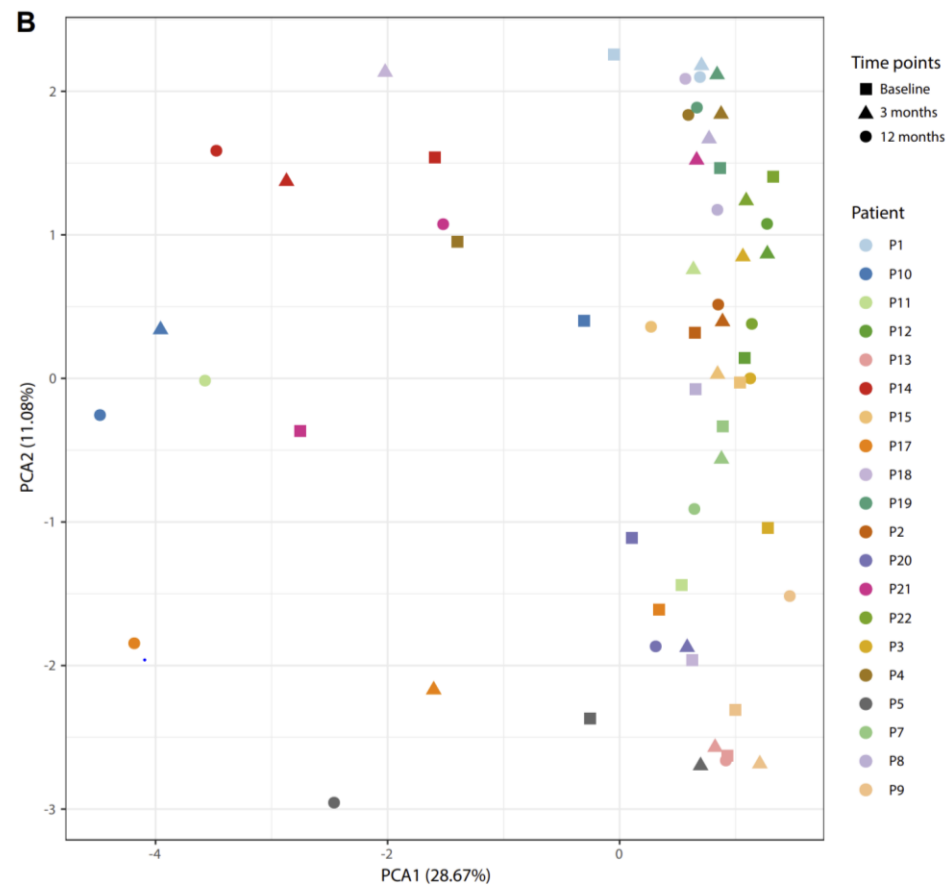

**Supplementary Figure 3**

Supplement: Supplemental Material [file KGMI_A_2157200_SM8536.zip › Supplementary information/Supplementary Figure 3 new.pdf]

### (A) Amoxicillin

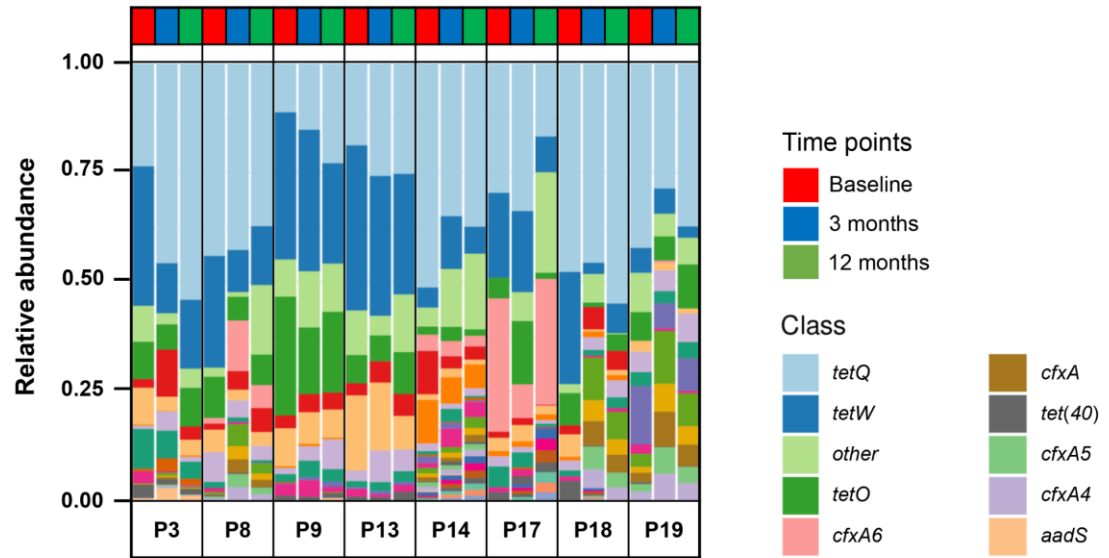

### (B) Placebo

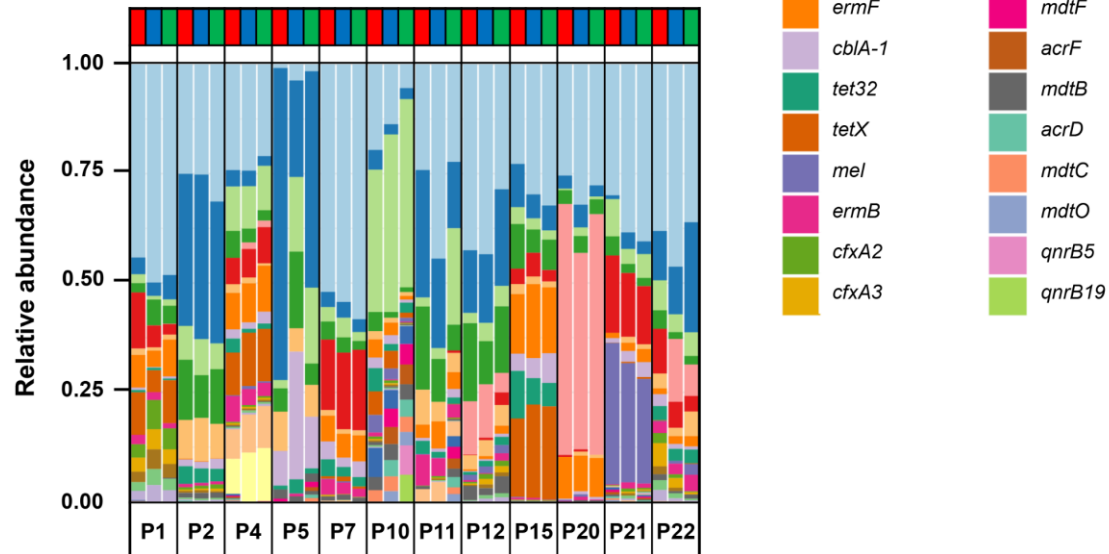

Supplementary Figure 4

Supplement: Supplemental Material [file KGMI_A_2157200_SM8536.zip › Supplementary information/Supplementary Figure 4.pdf]

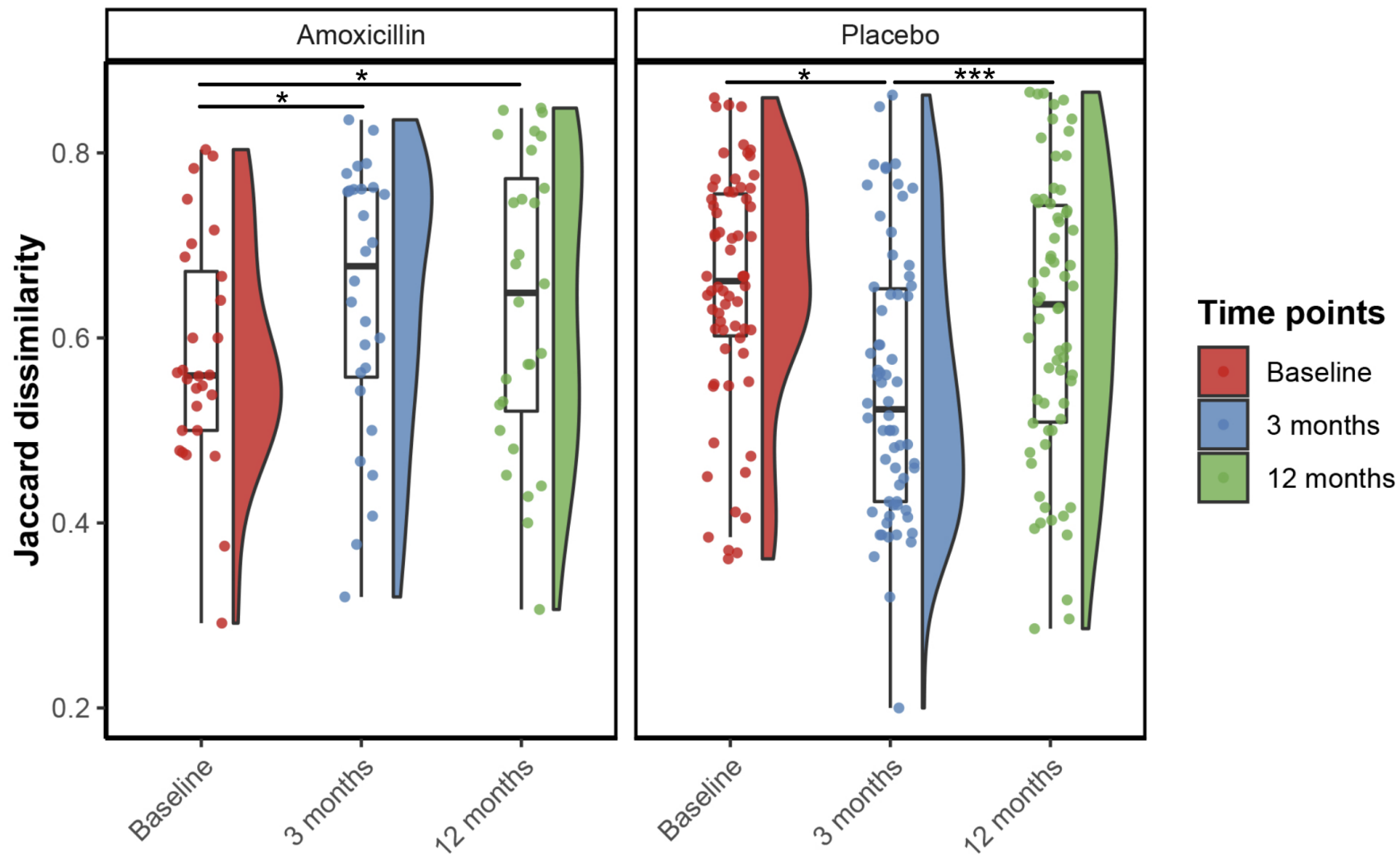

Supplementary Figure 5

Supplement: Supplemental Material [file KGMI_A_2157200_SM8536.zip › Supplementary information/Supplementary Figure 5.pdf]

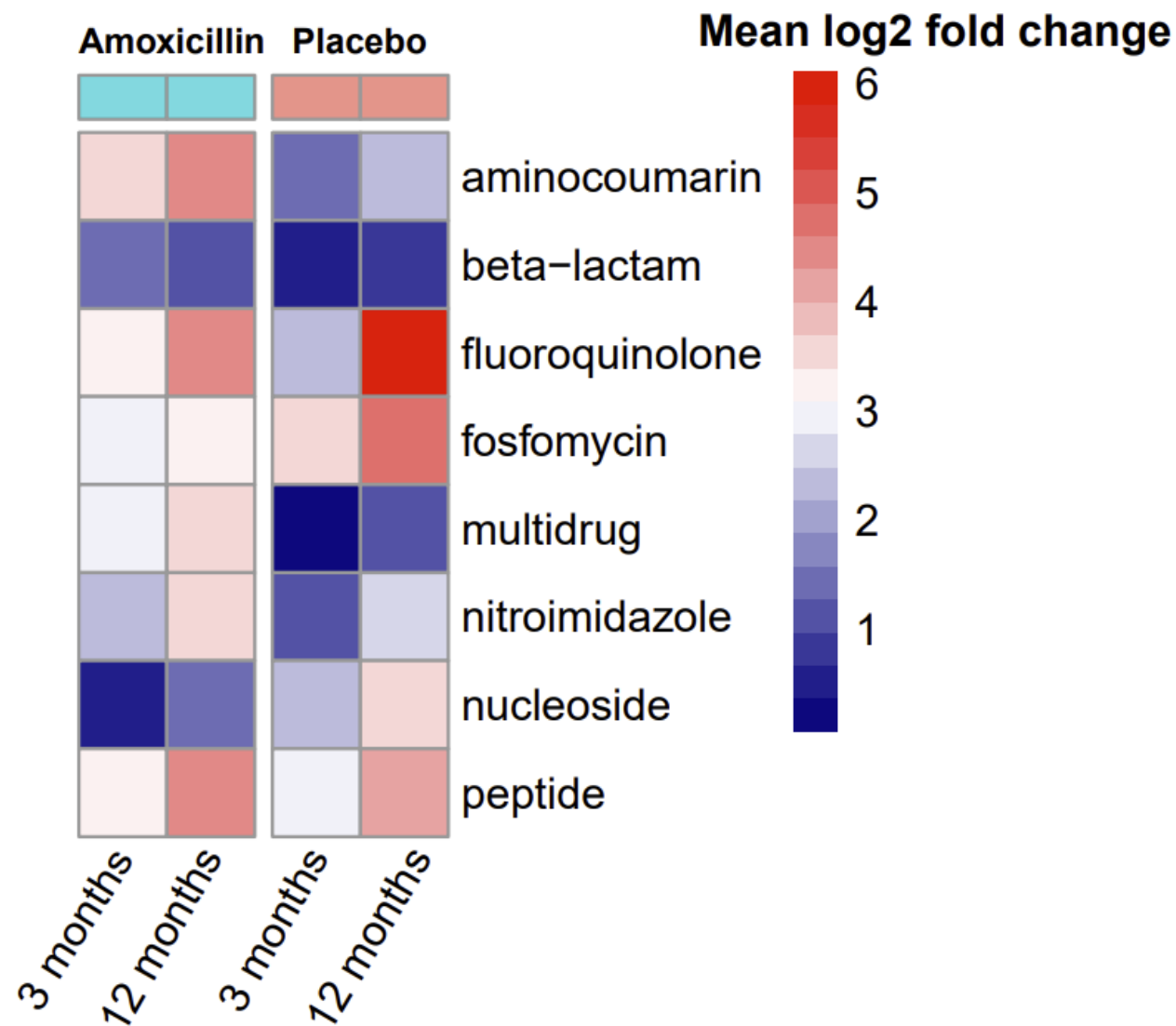

**Supplementary Figure 6**

Supplement: Supplemental Material [file KGMI_A_2157200_SM8536.zip › Supplementary information/Supplementary Figure 6.pdf]

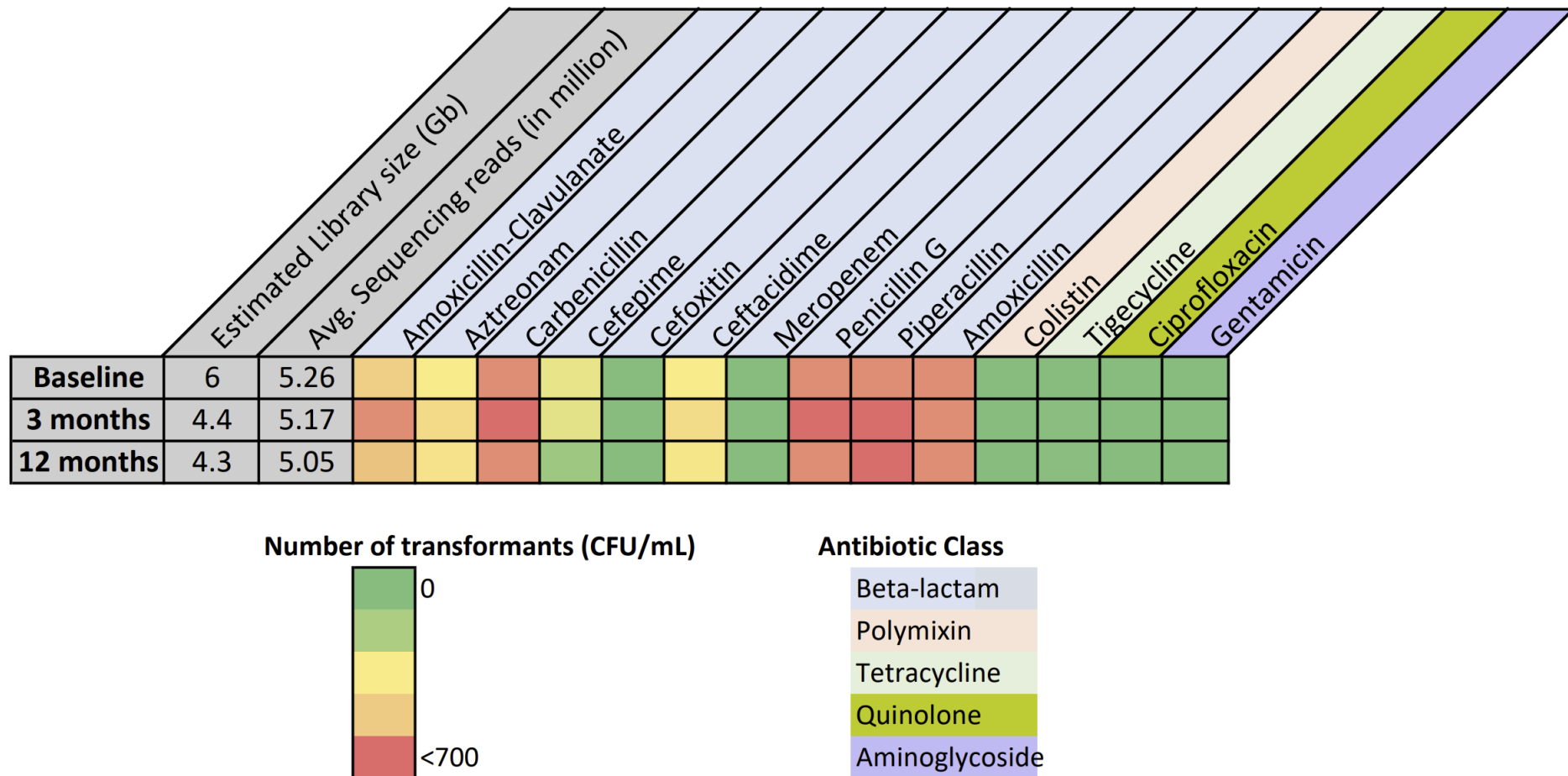

**Supplementary Figure 7**

Supplement: Supplemental Material [file KGMI_A_2157200_SM8536.zip › Supplementary information/Supplementary Figure 7.pdf]

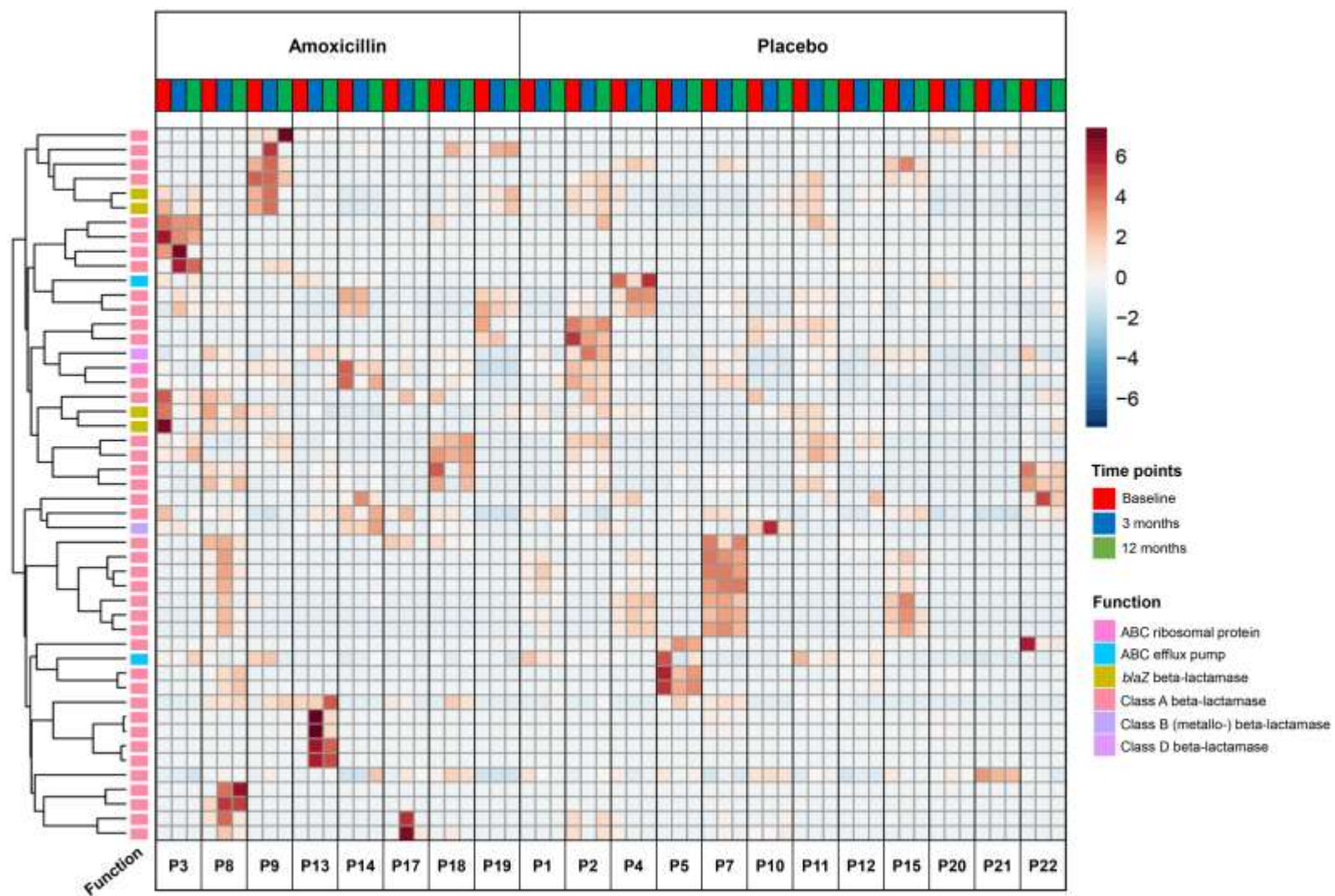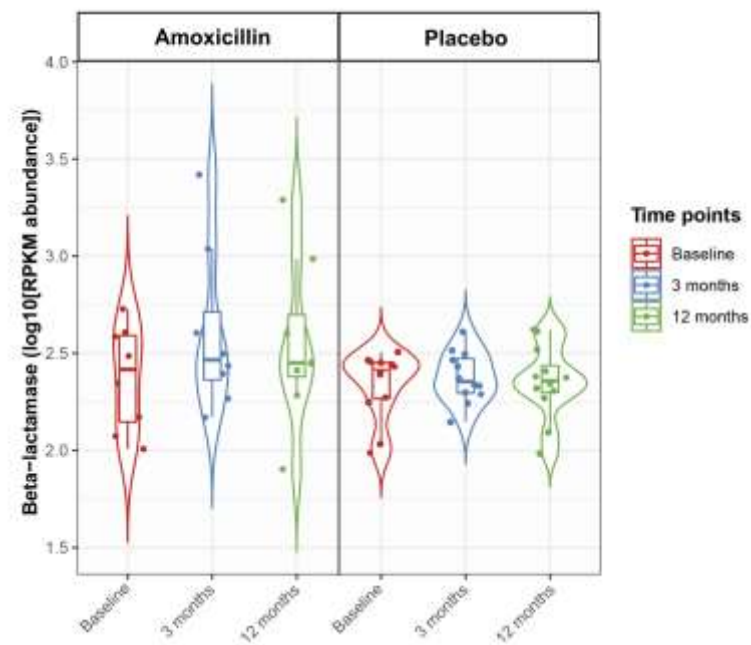

Supplementary Figure 8

Supplement: Supplemental Material [file KGMI_A_2157200_SM8536.zip › Supplementary information/Supplementary Figure 8.pdf]

**A**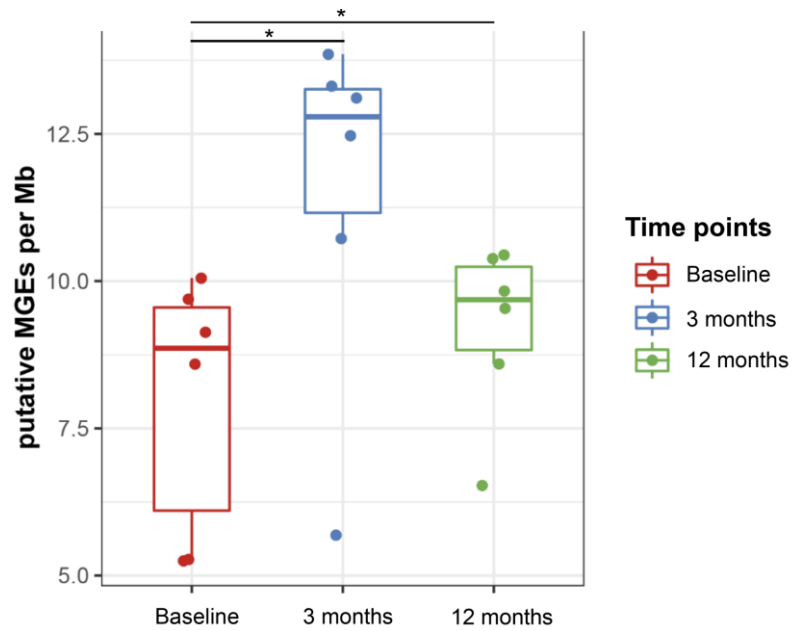**B**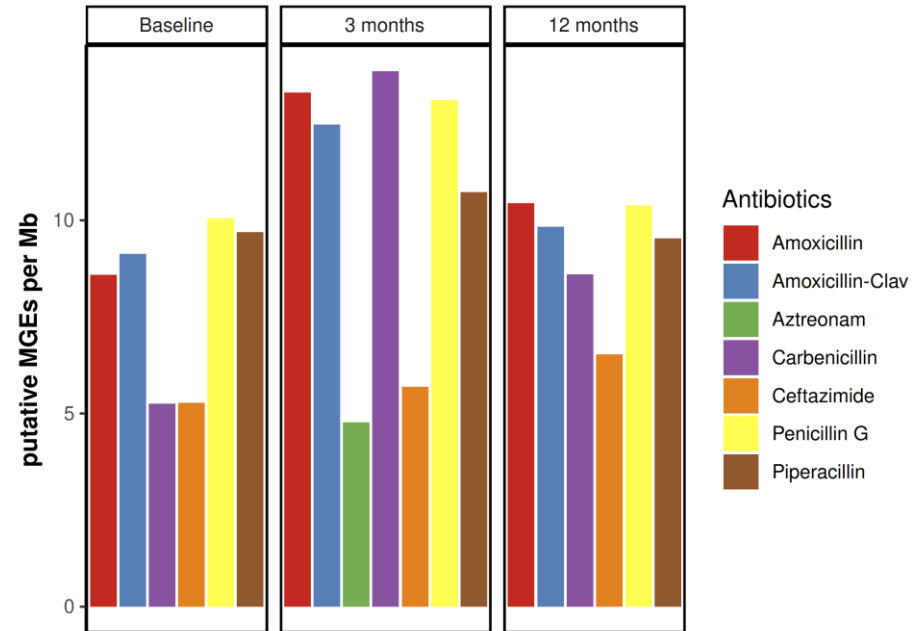

**Supplementary Figure 9**

Supplement: Supplemental Material [file KGMI_A_2157200_SM8536.zip › Supplementary information/Supplementary Figure 9.pdf]
